# Molecular and biochemical characterizations of a *Fasciola gigantica* retinoid X receptor-α isoform A (*Fg*RXRα-A)

**DOI:** 10.1038/s41598-024-63194-6

**Published:** 2024-05-29

**Authors:** Nattaya Torungkitmangmi, Pathanin Chantree, Salisa Chaimon, Parisa Prathaphan, Jittiporn Ruangtong, Amornrat Geadkaew-Krenc, Phornphan Sornchuer, Bumpenporn Sanannam, Nattaya Thongsepee, Viriya Pankao, Poom Adisakwattana, Pongsakorn Martviset

**Affiliations:** 1 Graduate Program in Biochemistry and Molecular Biology, Faculty of Medicine, Thammasat University, Pathum Thani, 12120 Thailand; 2 Department of Preclinical Science, Faculty of Medicine, Thammasat University, Pathum Thani, 12120 Thailand; 3 Thammasat University Research Unit in Nutraceuticals and Food Safety, Thammasat University, Pathum Thani, 12120 Thailand; 4 Graduate Program in Applied Biosciences, Faculty of Medicine, Thammasat University, Pathum Thani, 12120 Thailand; 5 Graduate Studies in Biomedical Sciences, Faculty of Allied Health Sciences, Thammasat University, Pathum Thani, 12120 Thailand; 6 Department of Helminthology, Faculty of Tropical Medicine, Mahidol University, Bangkok, 10400 Thailand

**Keywords:** *Fasciola gigantica*, Retinoid X receptor, *Fg*RXRα-A, 9-*cis* retinoic acid, Biochemistry, Drug discovery, Microbiology, Molecular biology, Diseases, Molecular medicine, Pathogenesis

## Abstract

Fascioliasis is a parasitic infection in animals and humans caused by the parasitic flatworm genus *Fasciola,* which has two major species, *F. hepatica* and *F. gigantica*. A major concern regarding this disease is drug resistance, which is increasingly reported worldwide. Hence, the discovery of a novel drug as well as drug targets is crucially required. Therefore, this study aims to characterize the novel drug target in the adult *F. gigantica*. In the beginning, we hypothesized that the parasite might interact with some host molecules when it lives inside the liver parenchyma or bile ducts, specifically hormones and hormone-like molecules, through the specific receptors, primarily nuclear receptors (NRs), which are recognized as a major drug target in various diseases. The retinoid X receptor (RXR) is a member of subfamily 2 NRs that plays multitudinous roles in organisms by forming homodimers or heterodimers with other NRs. We obtained the full-length amino acid sequences of *F. gigantica* retinoid X receptor-alpha (*Fg*RXRα-A) from the transcriptome of *F. gigantica* that existed in the NCBI database. The *Fg*RXRα-A were computationally predicted for the basic properties, multiple aligned, phylogeny analyzed, and generated of 2D and 3D models. Moreover, *Fg*RXRα-A was molecular cloned and expressed as a recombinant protein (r*Fg*RXRα-A), then used for immunization for specific polyclonal antibodies. The native *Fg*RXRα-A was detected in the parasite extracts and tissues, and the function was investigated by in vitro binding assay. The results demonstrated the conservation of *Fg*RXRα-A to the other RXRs, especially RXRs from the trematodes. Interestingly, the native *Fg*RXRα-A could be detected in the testes of the parasite, where the sex hormones are accumulated. Moreover, the binding assay revealed the interaction of 9-*cis* retinoic acid and *Fg*RXRα-A, suggesting the function of *Fg*RXRα-A. Our findings suggested that *Fg*RXRα-A will be involved with the sexual reproduction of the parasite by forming heterodimers with other NRs, and it could be the potential target for further drug development of fascioliasis.

## Introduction

*Fasciola gigantica* (*F. gigantica*), the liver fluke, is the major parasite causing fascioliasis or liver rot disease, especially in ruminants farmed in several continents, including Asia, Africa, and Oceania^1–3^. Regarding the economy, fascioliasis is a significant economic loss for farmers due to animal health problems, which reduce the quality and quantity of meat, wool, and milk products, as well as increase the cost of management^3–5^. Animals and humans can also be hosts of the parasite, which causes severe complications from intra- and extra-hepatic (ectopic) infections^2^. Fascioliasis is estimated to affect more than 700 million animals and about 2.4 million people worldwide and is regarded as a neglected tropical disease^6^. Triclabendazole is the most effective drug of choice for the treatment of fascioliasis in both animals and humans^7^. However, drug resistance has been increasingly reported^8–10^. For this reason, novel drugs and drug targets remain needed for discovery, but the parasite's complex life cycle and habitat are the most challenging factors^11^.

The nuclear receptors (NRs), also known as ligand-activated transcription factors, are the pharmacological potential targets of several drugs, whether they activate or inhibit the NR functions^12–14^. In humans, the NRs have been classified into several subfamilies based on their structure and specific ligands^12^. However, in parasitic organisms, the study on the NRs is very limited^15,16^. Generally, the NR structure comprises a conserved DNA-binding domain (DBD) located at the N-terminus and a ligand-binding domain (LBD) at the C-terminus. In addition, the LBD consists of a ligand-inducible transactivation function-2 (AF-2) domain that contributes to ligand specificity, while some NRs also have an amino-terminal ligand-independent activation function-1 (AF-1) domain which is unclear in function^14^. Moreover, the NRs could be activated mainly by lipophilic ligands, such as steroid hormones, vitamins, and retinoids, which then regulate downstream gene transcription^14^.

The life cycle of *F. gigantica*, which involves the host’s liver, where many hormones and bile salts are accumulating, makes us interested in the NRs in this parasite. We suspected some possibilities that NRs would be the key molecules that regulate the parasite biology inside the host body^17^. After getting an infection with *F. gigantica*, the newly excysted juvenile (NEJ) is released from the infective metacercaria, penetrates the intestinal wall, and finally becomes an adult in the liver parenchyma and major bile ducts^2^. At this stage, the parasite has the chance to interact with some molecules that could be specific ligands for NRs, such as bile salts (steroid-like molecules). It activates several intracellular signaling cascades through specific NRs. Our previous study reported the first NR found in *F. gigantica* (nuclear receptor subfamily 1; *Fg*NR1) with their molecular characteristics and primary function that could be activated by bile solution^17^. That finding, coupled with the genome project of *F. gigantica* released in the last few years^18,19^ leads us to find the other NRs in *F. gigantica* that could regulate a broader range of the parasite’s metabolisms.

The retinoid X receptor (RXR) is another interesting NR due to its functions involving several NRs by forming heterodimer^20^. Basically, the RXRs are classified in the subfamily 2 of nuclear receptors, which are subclassified into three groups, RXR-alpha (RXRα), RXR-beta (RXRβ), and RXR-gamma (RXRɤ), based on their structure, interacting molecules, and functions^21,22^. There are several NRs can form the heterodimer with RXR, especially subfamily 1 NR members, such as constitutive androstane receptor (CAR), farnesoid X receptor (FXR), bile acid receptor (BAR), liver X receptor (LXR), peroxisome proliferator–activated receptors (PPARs), pregnane X receptor (PXR), retinoic acid receptor (RAR), thyroid hormone receptor (TR), and vitamin D receptor (VDR). Delving into this aspect is not only to gain an in-depth understanding of the basic life cycle of *Fasciola* spp. Inhabiting the liver bile duct but identifying and characterizing RXRs in *F. gigantica* will increase the chance of novel drug development for fascioliasis. Therefore, this study aims to address the molecular characteristics, localization, and fundamental functions of *Fg*RXRα-A, the significant type of RXRs, that would influence the biochemical and physiological activities of the parasite when living inside the host that has never been reported before.

## Methods

### Ethics and biosafety statements

The animal study protocol was approved by the Institutional Care and Use Committee of Thammasat University (Protocol no. 012/2022 [date of approval: October 12th, 2022]). The production of polyclonal antibodies using ICR mice in this study was performed in the Laboratory Animal Center, Thammasat University (LAC-TU), and carried out strictly following the Guide for the Care and Use of Laboratory Animals of the National Institutes of Health (NIH), and reported in accordance with ARRIVE guidelines. For biosafety, all procedures used in this study were reviewed and approved by the Thammasat University Institutional Biosafety Committee (Approval no. 058/2562 [date of approval: May 14th, 2019], 007/2564 [date of approval: January 21st, 2021], and 007/2566 [May 30th, 2023]).

### Parasites

Adult *F. gigantica* parasites were retrieved from the bile ducts of naturally infected cattle sacrificed at the local slaughterhouses in Pathum Thani province, central Thailand. The worms were washed several times using 0.85% NaCl to remove debris and excessive bile contents. This study used both living and frozen adult worms.

### Bioinformatic analysis

The full-length cDNA encoding Retinoid X receptor-α of *F. gigantica* (*Fg*RXRα-A) and the deduced amino acid were obtained from the NCBI database (https://www.ncbi.nlm.nih.gov/; GenBank accession number: TPP55740.1)^23^. Firstly, the deduced amino acid sequence of *Fg*RXRα-A was aligned with RXRs reported from the other organisms to compare and confirm the conserved domains, including *Fasciola hepatica* (GenBank accession number: THD22775.1), *Schistosoma mansoni* (GenBank accession number: XP_018645908.1), *Clonorchis sinensis* (GenBank accession number: GAA56711.1), *Paragonimus heterotremus* (GenBank accession number: KAF5399899.1), *Bos taurus* (GenBank accession number: NP_001291272.1), and *Homo sapiens* (GenBank accession number: NP_002948.1) by using Clustral Omega^24^. Moreover, bioinformatics tools analyzed the *Fg*RXRα-A-deduced amino acid sequence for basic properties. The molecular mass and pI were calculated using EMBOSS Pepstats^25^. SignalP-5.0^26^, TMHMM server v. 2.0^27^, NetNGlyc 1.0, and NetOGlyc 4.0^28^ were used for signal peptide, transmembrane domain, N-, and O-glycosylation sites predictions, respectively. Disulfide bonds were forecasted by using the SCRATCH protein predictor^29^, whereas the protein family, conserved domains, sequence identity, and similarity were analyzed by using InterProScan from EMBL-EBI^30^ and Ident and Sim^31^. The phylogenetic tree was generated by MEGA11^32^ using the maximum likelihood method with 1000 bootstrap replications with more RXRs from trematodes, cestodes, nematodes, and mammals. The 2D and 3D structures of *Fg*RXRα-A were constructed using POLYVIEW-2D^33^ and SWISS-MODEL^34^, respectively. The amino acid sequences of all homologs used in this study are detailed in Supplementary data (Table S1).

### Molecular cloning of full-length *Fg*RXRα-A

Total RNA from adult *F. gigantica* was extracted using TRIzol reagent (Invitrogen, Carlsbad, CA, USA) following the instruction manual. The concentration of total RNAs was measured using NanoDrop™ 2000 Spectrophotometer (Thermo Fisher Scientific, Wilmington, Germany). The contaminating DNAs were removed by treating them with RNase-free DNaseI (Thermo Fisher Scientific, Vilnius, Lithuania) at 37 °C for 10 min. The complementary DNA (cDNA) was synthesized by reverse transcription using the RevertAid First Strand cDNA Synthesis Kit (Thermo Fisher Scientific, Vilnius, Lithuania) coupled with oligo(dT)18 primer. The full-length cDNA of *Fg*RXRα-A was amplified by PCR reaction using *Taq* DNA Polymerase (Thermo Fisher Scientific, Vilnius, Lithuania) in the mixture of *Taq* DNA Polymerase buffer, 0.2 mM of each dNTP, 2 mM MgCl_2_, 100 nM specific primers, and 0.5 µg of first strand cDNA as a template. The specific primers included forward primer (Fw) 5′-ATG AAT ATC AAT ATT TTG-3′ and reverse primer (Rv) 5′-TTC CAC TGA ACA GTT GT-3'. The PCR amplification consisted of initial denaturation at 95 °C for 5 min, followed by 35 cycles of denaturation, annealing, and extension at 95 °C for 1 min, 55 °C for 2 min, and 72 °C for 3 min, respectively. The final extension step was at 72 °C for 10 min. The PCR products were electrophoresed on 1% agarose gel using 1X TBE buffer at 100 V for 30 min and stained with ViSafe Red Gel Stain (Vivantis, Shah Alam, Malaysia). Following the manufacturer's instructions, the PCR product was extracted using the PureLink™ Quick Gel Extraction Kit (Invitrogen, Carlsbad, CA, USA). The purified PCR product was ligated to pGEM-T Easy Vector (Promega Corporation, Madison, WI, USA) and transformed using the MgCl_2_/CaCl_2_ heat shock method into XL1-Blue *Escherichia coli* competent cells. The cDNA sequence of *Fg*RXRα-A was verified for their correction by a DNA sequencing service (Solgent Co., Ltd., Daejeon, Republic of Korea).

### Production of *Fg*RXRα-A recombinant protein (r*Fg*RXRα-A) and thioredoxin (rTrx) fusion protein

The *Fg*RXRα-A cDNA was amplified by the exact condition above using the specific primers as follows: forward primer (Fw) 5′-GAG CTC CCC AGT TTT TCC AAC TTA C-3′ and reverse primer (Rv) 5′-AAG CTT TTC CAC TGA ACA GTT GT-3' that contained recognition sites of restriction endonucleases, *Sac*I and *Hind*III (underlined), respectively. The PCR amplicon was then purified and inserted into pGEM-T Easy Vector (Promega Corporation, Madison, WI, USA), then sub-cloned into pET32a(+) vector (Novagen, EMD Chemicals Inc., Darmstadt, Germany) that was used as an expression vector by digesting with *Sac*I and *Hind*III restriction enzymes (FastDigest™ restriction enzymes, Thermo Fisher Scientific, Lithuania). The recombinant pET32a(+)/*Fg*RXRα-A plasmid was chemically transformed into a BL21(DE3) *Escherichia coli* expression host, and the positive clones were selected by direct colony PCR as previously described^17^.

The recombinant protein of *Fg*RXRα-A was expressed simultaneously with the thioredoxin (Trx) fusion protein (r*Fg*RXRα-A + Trx) incorporated in the pET32a(+) expression vector. For the optimization of recombinant protein expression, the BL21(DE3) *E. coli* containing pET32a( +)/*Fg*RXRα-A PCR-positive clones were cultured in LB broth containing selective antibiotics and then induced with 1 mM isopropyl-d-1-thiogalactopyranoside (IPTG, Sigma-Aldrich, Saint Louis, MO, USA) at 37 °C for 1 to 3 h in shaking incubator. Bacterial cells were collected by refrigerated centrifugation at 4000 × *g* for 30 min at 4 °C. The pellet was lysed by using denaturing lysis buffer (8 M urea, 100 mM NaH_2_PO_4_, 10 mM Tris–Cl, pH 8.0) and verified for the overexpression and the solubility of r*Fg*RXR + Trx in a time-dependent manner by 12.5% SDS-PAGE. The expression condition of r*Fg*RXRα-A + Trx at 3 h after IPTG-induction was selected and used for producing and purifying the recombinant protein using the denaturing condition. The collected cells were lysed in the denaturing lysis buffer coupled with sonication at 70% amplitude for 2 min twice for five periods of 10–10 s pulse. The lysate was incubated in a rotary shaker for 1 h at 4 °C, centrifuged at 12,000×*g* for 30 min, and collected the supernatant. The collected supernatant was mixed with Ni-Sepharose^®^ high performance (Cytiva, Uppsala, Sweden) and incubated at 4 °C for 1 h with rotation, then loaded into a polypropylene column. The unbound flow-through was collected, and the beads were washed twice with wash buffer (8 M urea, 100 mM NaH_2_PO_4_, 10 mM Tris–Cl, pH 6.3). r*Fg*RXR + Trx was harvested by eluting with elution buffer (8 M urea, 100 mM NaH_2_PO_4_, 10 mM Tris–Cl, 250 mM imidazole, pH 8.0) for 4–5 fractions. All collected fractions were run on 12.5% SDS-PAGE and stained with Coomassie brilliant blue G-250 for evaluation. At the same time, the recombinant thioredoxin (rTrx) was produced from BL21(DE3) *E. coil* containing pET32a(+) and purified under native conditions as previously described^17,35,36^. The purified r*Fg*RXRα-A + Trx and rTrx were transferred to a micro-dialysis bag (Sigma-Aldrich, St. Louis, MO, USA) for dialysis against PBS, pH 7.4. Dialysis proceeded for 24 h at 4 °C with gentle stirring. The dialyzed r*Fg*RXRα-A + Trx and rTrx were concentrated using 3 kDa Vivaspin™ ultrafiltration spin columns (Cytiva, Buckinghamshire, UK), then verified by Western analysis with mouse anti-histidine tag antibody (Bio-Rad Laboratories Inc., Hercules, CA, USA). The concentrations of r*Fg*RXRα-A + Trx and rTrx were measured by BCA protein assay (Thermo Fisher Scientific, Rockford, IL, USA).

### Production of polyclonal antibodies against r*Fg*RXRα-A + Trx and rTdx

As previously described, the polyclonal antibodies against r*Fg*RXR + Trx and rTdx were produced in 6- to 8-week-old female ICR mice^17,37^. Pre-immune sera were collected, then the mice were immunized primarily with 100 µg of r*Fg*RXRα-A + Trx or rTrx that was mixed with TiterMax® Gold adjuvant (Sigma-Aldrich, St. Louis, MO, USA) using intraperitoneal injection. After two weeks, priming sera were collected, and the mice were immunized with 20 µg of r*Fg*RXRα-A + Trx or 50 µg of rTrx. Two weeks later, the 1st-boosted sera were collected, and the mice were repeatedly immunized. The final sera were collected after two weeks of the injection by cardiac puncture.

The titers of anti-r*Fg*RXRα-A + Trx and anti-rTrx polyclonal antibodies were determined by indirect ELISA. A 96-well microtiter plate was coated with 150 ng r*Fg*RXRα-A + Trx or rTdx in coating buffer, pH 9.6 (30 mM Na_2_CO_3_, 75 mM NaHCO_3_) and incubated overnight at 4 °C in a humid chamber. After incubation, the wells were washed with dH_2_O thrice; then, the non-specific bindings were blocked using 0.25% Bovine Serum Albumin (Sigma-Aldrich, St. Louis, MO, USA) at room temperature for 30 min. The unbound proteins were removed by washing with dH_2_O thrice. The sera were serial-diluted in antibody diluents (0.25% BSA in 10 mM PBS, pH 7.4) and added to the wells for 100 μl each. All dilutions of sera were assayed in duplicate. The plate was incubated at 37 °C for 1 h and washed thrice. Goat anti-mouse IgG (H + L) HRP antibody (Sigma-Aldrich, St. Louis, MO, USA) at dilution of 1:10,000 was added to the reaction and incubated for 1 h at 37 °C, and 1-Step™ Ultra TMB-ELISA Substrate Solution (Sigma-Aldrich, St. Louis, MO, USA) was added. The colorimetric reaction proceeded in the dark for 1 h and was stopped by adding HCl. The absorbance was measured at 492 nm on a plate reader, and the positive reactions were determined based on the cut-off value calculated using the change-point analysis method^38^.

### Preparation of crude worm antigens (CWA) and excretory-secretory (ES) products

The crude worm antigens (CWA) were prepared using the homogenization method as previously described^37^. Adult *F. gigantica* were homogenized in homogenization buffer (0.01 M PBS, pH 7.4 containing 1% Triton X-100, 2 mM PMSF, 5 mM iodoacetamide, and 1 mM EDTA). The homogenate was centrifuged at 12,000×*g* for 30 min at 4 °C, and the supernatant was collected as soluble CWA (CWA-S). The pellet was then resuspended in solubilizing buffer (50 mM Tris–Cl, pH 8.0, and 3% SDS) and incubated at 37 °C for 1 h. The supernatant collected from this step is called insoluble CWA (CWA-I).

The excretory-secretory (ES) products were prepared from freshly collected live adult worms. The parasites were cultured in PBS, pH 7.4, at 37 °C under a 5% CO_2_ atmosphere for 4 h with gentle shaking. Parasite eggs and insoluble particles were removed by centrifugation at 4000×*g* for 30 min. The ES products were concentrated using 3 kDa Vivaspin™ ultrafiltration spin columns (Cytiva, Buckinghamshire, UK). The concentrations of CWA and ES products were determined by Pierce™ BCA Protein Assay Kits (Thermo Scientific™).

### Western analysis

r*Fg*RXRα-A-Trx (100 ng), rTrx (100 ng), CWA-S (12 µg), CWA-I (20 µg), and ES products (6.7 µg) of *F. gigantica* were size separated on 12.5% SDS-PAGE and transferred onto a nitrocellulose membrane (Cytiva, Buckinghamshire, UK) by semi-dry blotting (Invitrogen™ Power Blotter, Thermo Fisher Scientific, Carlsbad, CA, USA). After blotting, the membrane was soaked in a 5% skim milk blocking solution (Sigma Aldrich, Darmstadt, Germany) and subsequently incubated with mouse anti-r*Fg*RXRα-A, anti-rTrx, or preimmunized sera in the dilution of 1:400 in antibody diluent (1% BSA in PBS, pH 7.4) at 4 °C, for overnight. The membrane was washed thrice with PBST (1X TBS, pH 7.5, containing 0.05% Tween 20). The HRP conjugated anti-mouse IgG (H + L) secondary antibody (Invitrogen, Thermo Fisher Scientific, Carlsbad, CA, USA) was used. The membrane was washed with PBST three times and incubated with a 3,3’,5,5’-Teteramethyl benzidine (TMB) substrate (Sigma-Aldrich, St. Louis, MO, USA) until the signals developed.

### Preparation of paraffin-embedded tissue sections and immunolocalization

The tissue section and immunolocalization were handled as previously described^17^. Paraffin-embedded tissue was prepared to detect the native *Fg*RXRα-A in the parasite tissues. The fresh worms were cut into small pieces and fixed with cold 4% paraformaldehyde for 1 h at 4 °C. The fixative agent was replaced with an ethanol alcohol series from 50 to 100%, followed by fresh xylene, xylene/paraplast, and pure paraplast. The parasites were embedded in paraffin in a mold and then cut into an 8 µm thickness section using a microtome (Leica RM 2235, Nussloch, Wetzlar, Germany). The slide-mounted sections were stored dry and dust-free for further experiments.

To determine *Fg*RXRα-A in the parasite tissues, mouse polyclonal antibodies against r*Fg*RXRα-A + Trx or rTrx were used. The tissue sections were re-hydrated by removing the paraffin with fresh xylene twice for 10 min each and then passing through an alcohol series from 100 to 70% ethanol. The tissue sections were rinsed with dH_2_O and proceeded to the epitope retrieval step. The epitopes were retrieved by immersing the sections in epitope retrieval solution (10 mM Na_3_C_6_H_5_O_7_, pH 6.0 with 0.05% Tween® 20), then heated in a microwave oven for 5 min. The tissue sections were incubated with glycine blocking solution (0.1% glycine in PBS, pH 7.4) for 30 min at room temperature and 4% BSA in PBS, pH 7.4 for 1 h at room temperature to prevent unspecific bindings. The internal peroxidase was blocked with 3% hydrogen peroxide for 30 min. Mouse anti-r*Fg*RXRα-A, anti-rTrx, or preimmunized sera were added to the tissue sections at the dilution of 1:50 in antibody diluent (1% BSA in PBS, pH 7.4) and incubated at 4 °C for overnight in a humid chamber. The next day, the tissue sections were washed three times with washing buffer before adding Rabbit anti-mouse IgG (H + L) secondary antibody, Biotin (Thermo Fisher Scientific, St. Louis, MO, USA), for 30 min at room temperature. The tissue sections were washed three times with washing buffer and incubated with AEC substrate (Thermo Fisher Scientific, Rockford, IL, USA) in the dark until the signals could be observed. The reaction was stopped with dH_2_O. The tissue sections were investigated under a light microscope.

### Construction of pFN26A (BIND) hRluc-neo Flexi®/*Fg*RXRα-A-LBD recombinant plasmid

The ligand binding domain of *Fg*RXRα-A (*Fg*RXRα-A-LBD) was amplified by using the full-length *Fg*RXRα-A as a template, and the specific primers as follows: forward primer (Fw) 5′-GCG ATC GCC CAC CAC CGT TAA GTC TAG CT-3′ and reverse by primer (Rv) 5′-GCG GCC GCG CCA TGC TCG ACC AAA TTA A-3′ which incorporated with *Not*I and *Sgf*I recognition sites (underlined), respectively. The PCR amplification was carried out as described above using initial denaturation at 95 °C for 5 min, followed by 35 cycles of denaturation at 95 °C for 1 min, annealing at 60 °C for 1 min, extension at 72 °C for 1 min, and one cycle of a final extension at 72 °C for 10 min. The PCR product was purified and ligated into pGEM-T Easy vector (Promega Corporation, Madison, WI, USA) and chemically transformed into XL1-Blue *E. coli* competent cells using the heat shock method, then verified the sequence correctness by DNA sequencing.

The *Fg*RXRα-A-LBD fragment was digested from pGEM-T Easy by using *Not*I and *Sgf*I restriction enzymes and eluted from agarose gel using PureLinkTM Quick Gel Extraction Kit (Invitrogen, Carlsbad, CA, USA). The *Fg*RXRα-A-LBD fragment was subcloned into pFN26A (BIND) hRluc-neo Flexi® Vector (Promega, Madison, WI, USA). The recombinant plasmid was chemically transformed into XL1-Blue *E. coli* competent cells using heat shock. As previously mentioned, the positive transformants were selected by direct colony PCR using a specific primer.

### Establishment of HEK293/pGL4.35[luc2P/9XGAL4UAS/Hygro] stable cell line

HEK293 cell line was purchased from the American Type Culture Collection (ATCC) (Manassas, VA, USA). HEK293 cells were cultured in DMEM medium supplemented with 10% fetal bovine serum at 37 °C in a humidified atmosphere at 5% CO_2_. A stable cell line containing 9XGAL4UAS-luc2P was generated by lipid transfection of HEK293 with pGL4.35 [luc2P/9XGAL4UAS/Hygro] (Promega, Madison, WI, USA) using Lipofectamine® 3000 (Invitrogen, Carlsbad, CA, USA) following the manufacturer’s protocol in Opti-MEM™ serum-free medium (Gibco™, Dublin, Ireland). A hygromycin-resistant population was selected by adding 500 ug/ml hygromycin to the culture medium and continuously cultured for two weeks in the maintained concentration of hygromycin. The stable cell line was cultured until the confluence reached 80%, then sub-cultured and expanded.

### Functional assay

The functional assay was done following the previous report with a few modifications^39^. HEK293/pGL4.35[luc2P/9XGAL4UAS/Hygro] stable cells were seeded into a 96-well culture plate at the density of 20,000 cells/well. The cells were cultured in DMEM medium supplemented with 20% fetal bovine serum at 37 °C with a humidified atmosphere at 5% CO_2_ for 48 h. The pFN26A (BIND) hRluc-neo Flexi®/*Fg*RXRα-A-LBD recombinant plasmid was purified by PureYeild™ plasmid miniprep system (Promega, Madison, WI, USA) and transiently transfected into the HEK293/pGL4.35[luc2P/9XGAL4UAS/Hygro] stable cells using Lipofectamine® 3000 (Invitrogen, Carlsbad, CA, USA) and then cultured for further 24 h. After transfection, the transfecting medium was removed, and the cells were exposed to 1 mM 9-*cis* retinoic acid (9-*cis* RA), 1% bovine bile solution, or plain Opti-MEM™ medium at 37 °C for 24 h. The pFN26A (BIND) hRluc-neo Flexi® vector was used as a background control. The transfected cells were assayed 24 h after exposure, and the luciferase signals were observed every 5 min after the reactions were stopped using the Dual-Glo® Luciferase Assay System (Promega, Madison, WI, USA) following the manufacturer’s instruction manual. The students' *t*-test was used to verify the statistical analysis between the test groups. A *p*-value < 0.05 was considered a significant difference.

## Results

### General properties, molecular characteristics, and computational modeling of *Fg*RXRα-A

**Figure 1:**
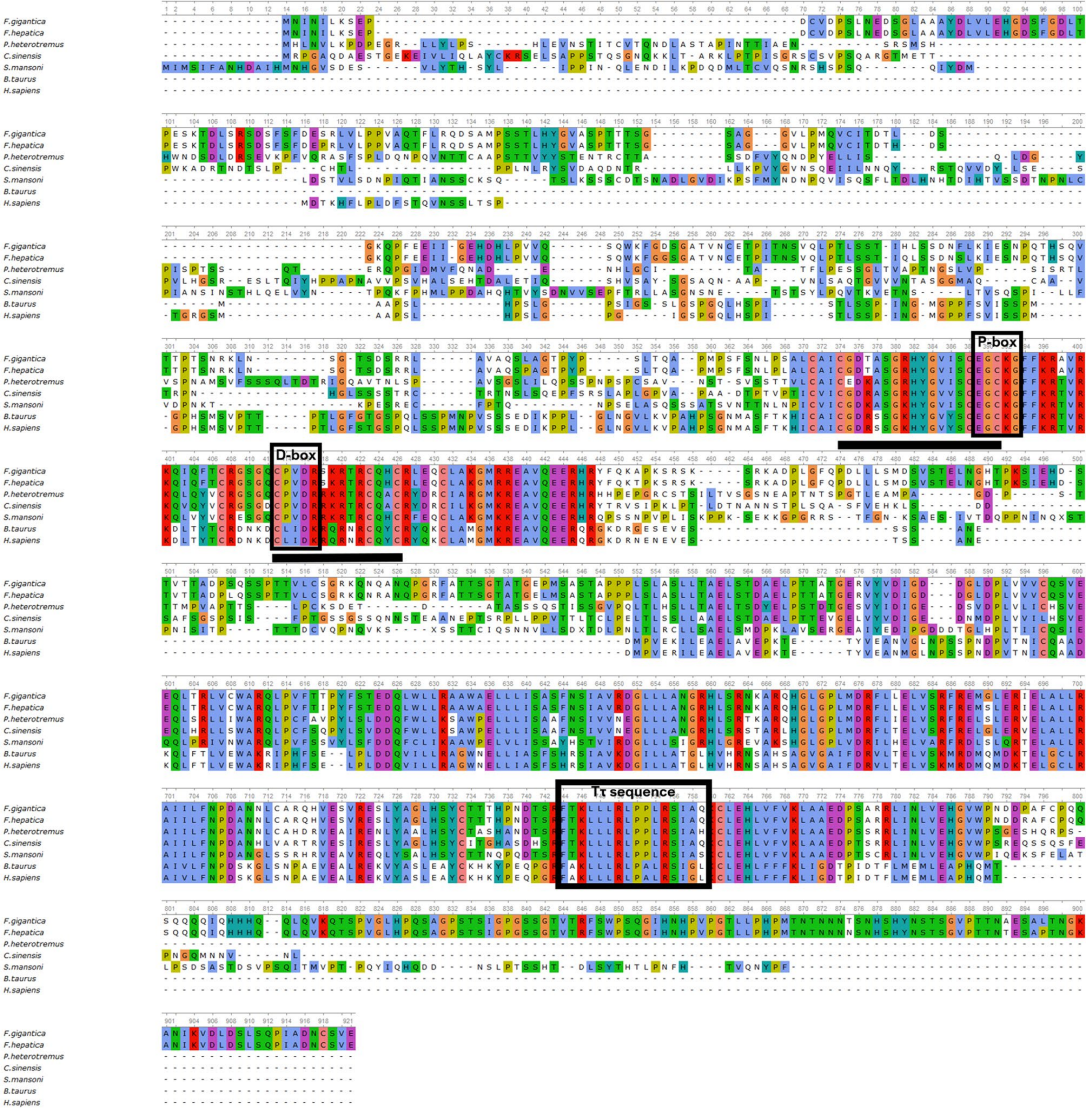
Multiple alignments of *Fg*RXRα-A with homologs from other flatworms and mammals showing the conserved domains in both DNA binding domain (DBD), P-box and D-box located in the zinc finger regions (black underlines), and ligand binding domain (LBD) with Tτ sequence (nuclear receptor signature sequence).

The conserved motifs and consensus residues of the *Fg*RXRα-A obtained from the database were identified by the multiple sequence alignments compared with the RXR homologs including *F. hepatica* (*Fh*RXRα-A),* S. mansoni* (*Sm*RXR), *C. sinensis* (*Cs*RXRα), *P. heterotremus* (*Ph*RXRα-A), *B. taurus* (*Bt*RXRα), and *H. sapiens* (*Hs*RXRα-A). The multiple alignments were done in the DNA binding domain (*Fg*RXRα-A-DBD) and the ligand binding domain (*Fg*RXRα-A-LBD). The result demonstrated that *Fg*RXRα-A is highly conserved to the RXR subfamily of both DBD and LBD (Fig. 1). Sequence alignment of the *Fg*RXRα-A-DBD revealed two conserved zinc-finger regions at positions 374–391 and 413–426, including two functional domains, P-box and D-box. Moreover, the *Fg*RXRα-A-LBD demonstrated the nuclear receptor-ligand binding domain's (NR-LBD) signature sequence (Tτ sequence) at positions 744–759. However, the *Fg*RXRα-A-LBD has the highest conservation to *Fh*RXRα-A, derived from *F. hepatica*, the closely related organism. Moreover, the *Fg*RXRα-A-LBD revealed higher conservation to the RXRs from the flatworm parasites, including *Sm*RXR, *Cs*RXRα, and *Ph*RXRα-A than the mammals (*Bt*RXRα), and human (*Hs*RXRα-A).

For the general characteristics, the *Fg*RXRα-A coding sequence (CDS) comprised 1533 nucleotides, which translated into 511 amino acids. The deduced amino acids were predicted for molecular weight (MW) and isoelectric point (PI) using EMBOSS Pepstats, revealing an MW of 60.355 kDa and a PI of 8.3669. The signal peptide prediction revealed that *Fg*RXRα-A is absent of signal peptide. Moreover, *Fg*RXRα-A does not contain a transmembrane domain. A total of 5 potential N-glycosylation sites and 62 potential O-glycosylation sites were predicted. The SCRATCH protein predictor demonstrated that *Fg*RXRα-A has 17 cysteine residues, which formed six disulfide bonds. All general properties of *Fg*RXRα-A are shown in Supplementary Data Table S2.

**Figure 2:**
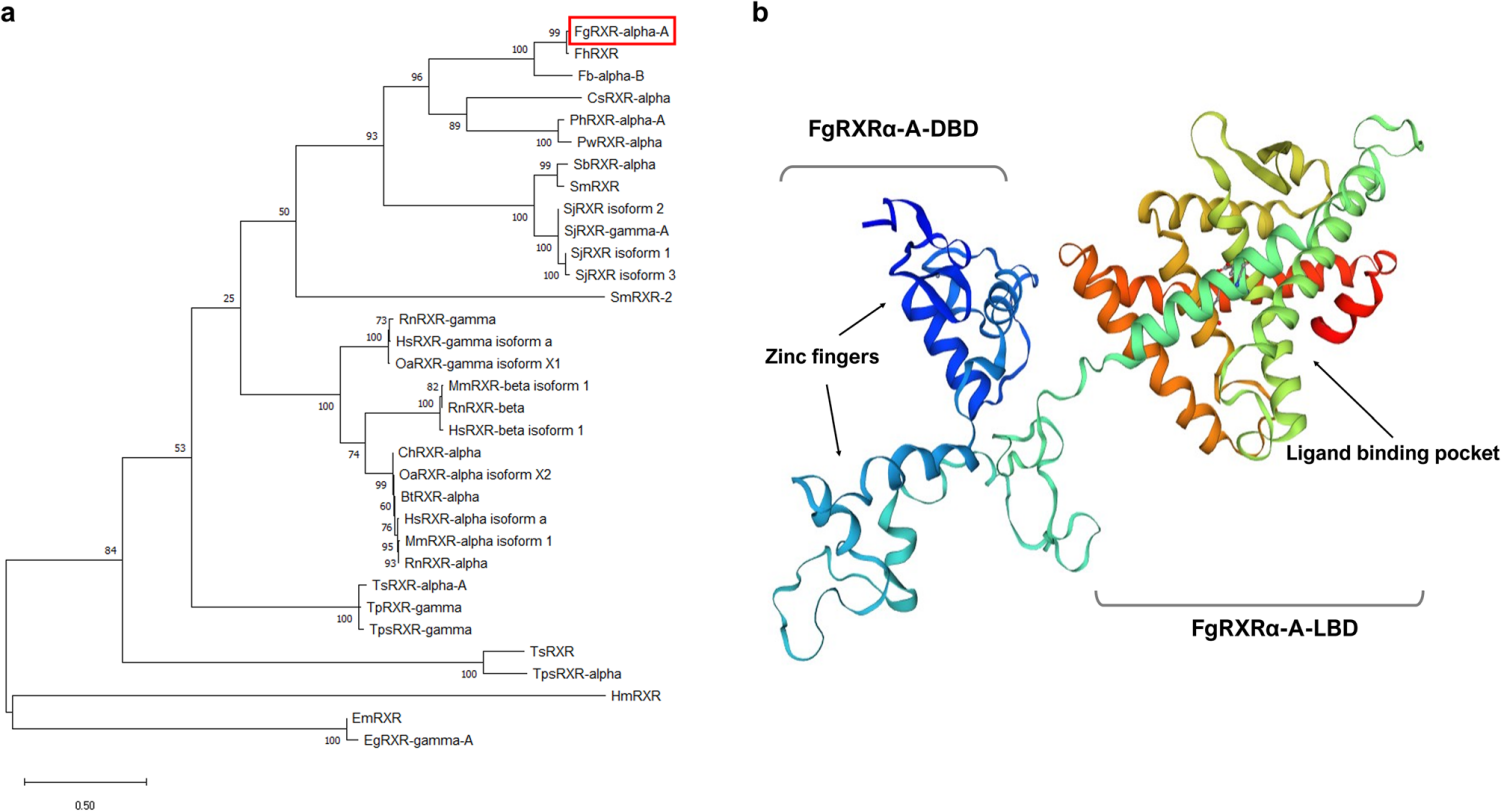
(**a**) Phylogenetic analysis demonstrates the conservation of *Fg*RXRα-A to other homologs, particularly the cluster of RXRs from flatworms. (**b**) The 3D structure of *Fg*RXRα-A demonstrates the DBD (with two zinc finger proteins) and LBD domains (with ligand binding pocket).

For obtaining the evolutionary data of *Fg*RXRα-A, more members of the RXR subfamily were included for generating the phylogenetic tree. The result demonstrated that the *Fg*RXRα-A was closely related to *Fh*RXR (*Fasciola hepatica* RXR) and *Fb*RXRα-B (*Fasciolopsis buski* RXR alpha isoform B) with a very high score and incorporated in the same branch. Additionally, the other flatworm RXRs, including *Cs*RXRα (*Clonorchis sinensis* RXR alpha), *Ph*RXRα-A (*Paragonimus heterotremus* RXR alpha isoform A), *Pw*RXRα (*Paragonimus westermani* RXR alpha), *Sb*RXRα (*Schistosoma bovis* RXR alpha), *Sm*RXR (*Schistosoma mansoni* RXR), *Sj*RXR2 (*Schistosoma japonicum* RXR isoform 2), *Sj*RXRɤ-A ((*Schistosoma japonicum* RXR gamma isoform A), *Sj*RXR1 (*Schistosoma japonicum* RXR isoform 1), and *Sj*RXR3 (*Schistosoma japonicum* RXR isoform 3) demonstrated high evolutionary score, except *Sm*RXR2 (*Schistosoma mansoni* RXR isoform 2) which demonstrated the unrelated evolution to the others. In contrast, the nematode, cestode, and mammal RXRs illustrated the lower scores, which indicated less evolutionary relations (Fig. 2a). However, the comparative heatmap provides more detailed information by showing the similarity and identity percentages of *Fg*RXRα-A with other reported RXR homologs (Fig. S1a). The identity heatmap demonstrated that even though the evolutionary score from the phylogenetic tree was very high, the identity was not 100%. The *Fg*RXRα-A and *Fh*RXR showed 97.79% similarity. Moreover, in relation to the evolutionary phylogeny, the heatmap demonstrated that the RXRs from flatworm parasites have high similarity scores that are less conserved than the mammal RXRs.

For the *in-silico* modeling, the *Fg*RXRα-A was predicted for 2D structure using the POLYVIEW-2D. The predicted structure consisted of 6 β-strands, 11 α-helices, and 18 coils (Fig. S1b). The 3D structure of *Fg*RXRα-A was generated using the I-TASSER. The crystal structure of liganded hRXRα/hLXRβ (human liver X nuclear receptor beta) heterodimer was utilized as a template for the 3D structure modeling. The result demonstrated that *Fg*RXRα-A 3D structure closely resembled hRXR-alpha/hLXR-beta heterodimer. The *Fg*RXRα-A structure included a DNA binding domain (DBD) on the left side with two zinc finger motifs (blue) comprising a beta-hairpin coupled with two helices that specifically recognize the target DNA. On the other side of the model was the ligand-binding domain (LBD). It comprises several helices and another hairpin that forms the ligand-binding pocket. The 3D structure of *Fg*RXRα-A is shown in Fig. 2b.

### *Fg*RXRα-A has been successfully cloned and produced as a recombinant protein

**Figure 3:**
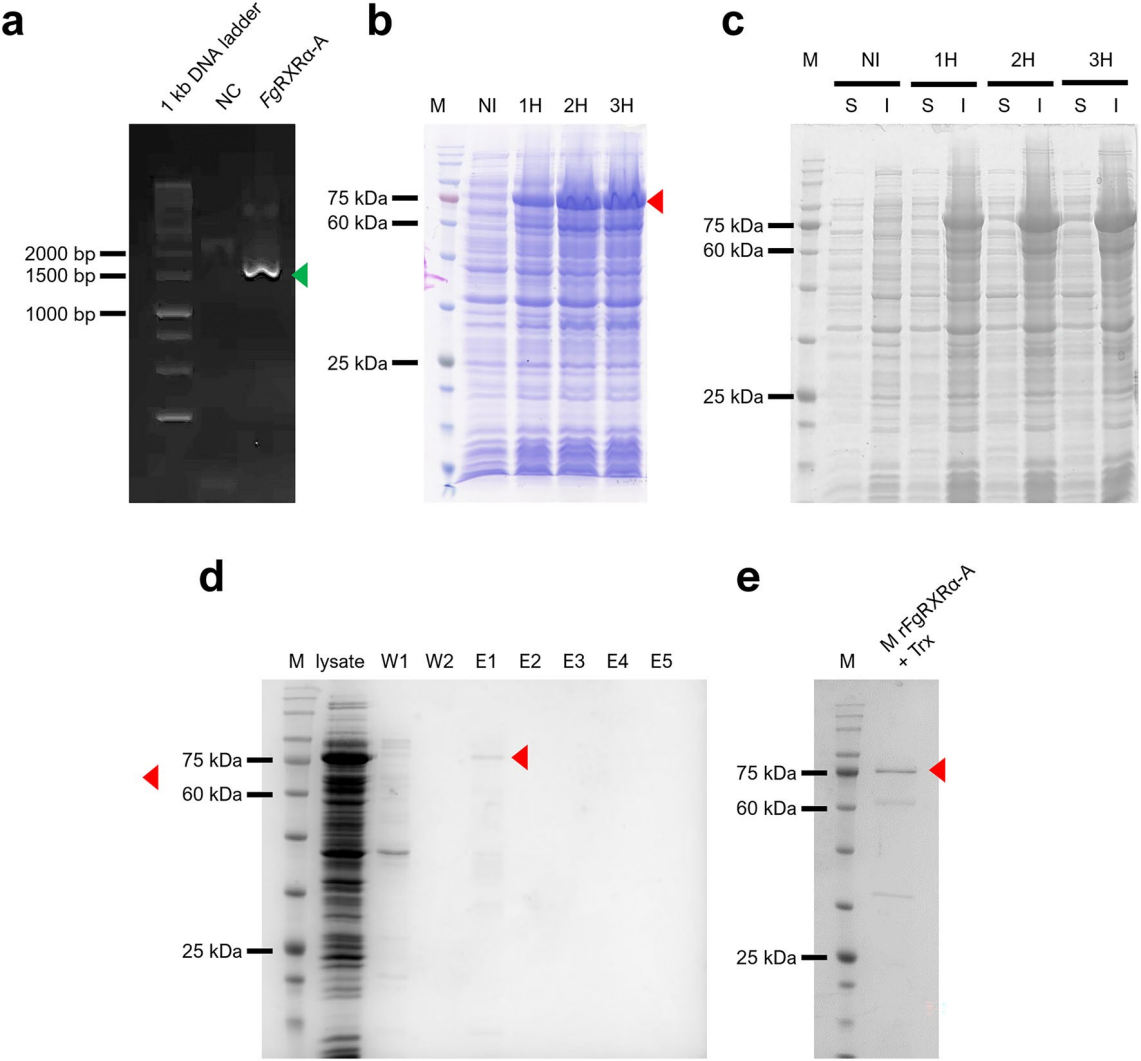
Molecular cloning and production of rFgRXRα-A. (**a**) The PCR amplicon of *Fg*RXRα-A at the expected size (1533 bp) (green arrow). (**b**) Timepoint analysis of r*Fg*RXRα-A with Trx fusion protein (red arrow) after 1 mM IPTG induction for 1 (1H), 2 (2H), and 3 (3H) shows overexpression at the expected size (74 kDa) in every collected timepoint. M = Tricolor Broad Range Prestained Protein Ladder, Vivantis, Malaysia; NI = non-induced bacteria. (**c**) The evaluation of solubility of r*Fg*RXRα-A + Trx shows the expression in the inclusion bodies at all collected time points (1H–3H). S = soluble fraction and I = insoluble fraction. (**d**) Purified r*Fg*RXRα-A + Trx under the denaturing condition. W1 = wash fraction 1; W2 = wash fraction 2; E1–E5 = elution fractions 1 to 5. (**e**) Concentrated r*Fg*RXRα-A + Trx (red arrow).

The *Fg*RXRα-A cDNA fragment was successfully synthesized (Fig. 3a) and cloned into the expression vector. The r*Fg*RXRα-A + Trx was overexpressed in all collected time points (1 – 3 h after IPTG induction) at the expected size (74 kDa) (Fig. 3b) and included in the inclusion bodies (Fig. 3c). The r*Fg*RXRα-A + Trx was purified under the denaturing condition (Fig. 3d) and the concentrated r*Fg*RXRα-A + Trx is shown in Fig. 3e.

### The native *Fg*RXRα-A could be detected in the parasite crude extract (CWA) and highly expressed in the testes of the adult parasite

**Figure 4:**
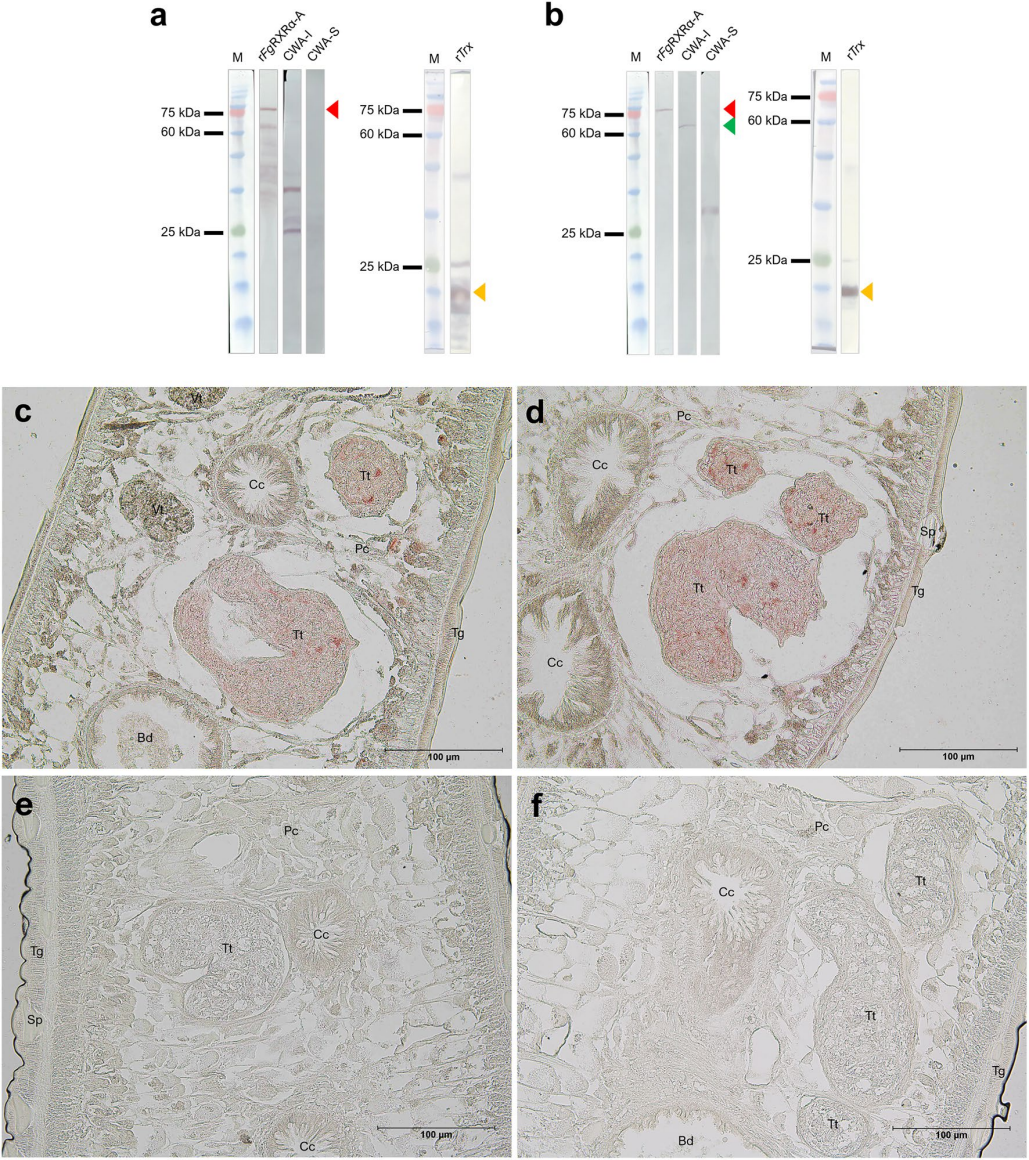
The detection of native *Fg*RXRα-A. Western analysis of r*Fg*RXRα-A, crude worm antigen soluble fraction (CWA-I), crude worm antigen insoluble fraction (CWA-S), and rTrx with anti-rTrx polyclonal antibodies (**a**), and anti-r*Fg*RXRα-A polyclonal antibodies (**b**). The expected sizes are indicated by red arrows (r*Fg*RXRα-A; 74 kDa), green arrow (native *Fg*RXRα-A; 60 kDa), and yellow arrows (rTrx). M = Tricolor Broad Range Prestained Protein Ladder, Vivantis, Malaysia. The immunolocalization using anti-r*Fg*RXRα-A antisera (**c, d**) indicates the expression of native *Fg*RXRα-A in the testes (Tt) (reddish signals). In contrast, preimmune sera (**e**) and anti-rTrx antisera (**f**) show precise negative results. Bd = bladder, Cc = caecum, Pc = parenchyma, Sp = spine, Tg = tegument, Tt = testes, and Vt = vitelline.

The Western analysis was done with crude worm antigens, both soluble (CWA-sol) and insoluble (CWA-insol) fractions, ES products, and r*Fg*RXRα-A + Trx using preimmunized sera, anti-r*Fg*RXRα-A + Trx, and anti-Trx antibodies. The preimmunized sera demonstrated negative detections in all antigens (CWA-sol, CWA-insol, ES products, and r*Fg*RXRα-A + Trx) (Fig. S2). The anti-Trx antibodies could detect only the r*Fg*RXRα-A + Trx at the predicted size (74 kDa) (Fig. 4a). The anti- r*Fg*RXRα-A + Trx detected r*Fg*RXRα-A + Trx at the expected size (74 kDa). Moreover, the anti-r*Fg*RXRα-A + Trx demonstrated the positive band in the insoluble crude worm fraction (CWA-I) at the predicted size of native *Fg*RXRα-A (approximately 60 kDa) (Fig. 4b). However, in the CWA-sol, one positive band at the lower size (between 25 and 35 kDa) was found in every repeated experiment without explanation. For the immunolocalization of the native *Fg*RXRα-A in the parasite tissue, the result of anti-r*Fg*RXRα-A + Trx demonstrated that *Fg*RXRα-A was highly expressed in the testes of the adult parasite (Fig. 4c, d). In contrast, the preimmunized sera and anti-Trx illustrated negative signals in all organs (Fig. 4e, f).

### 9-*cis *retinoic acid (RA) activated the *Fg*RXRα-A function by enhancing the luciferase activity

**Figure 5:**
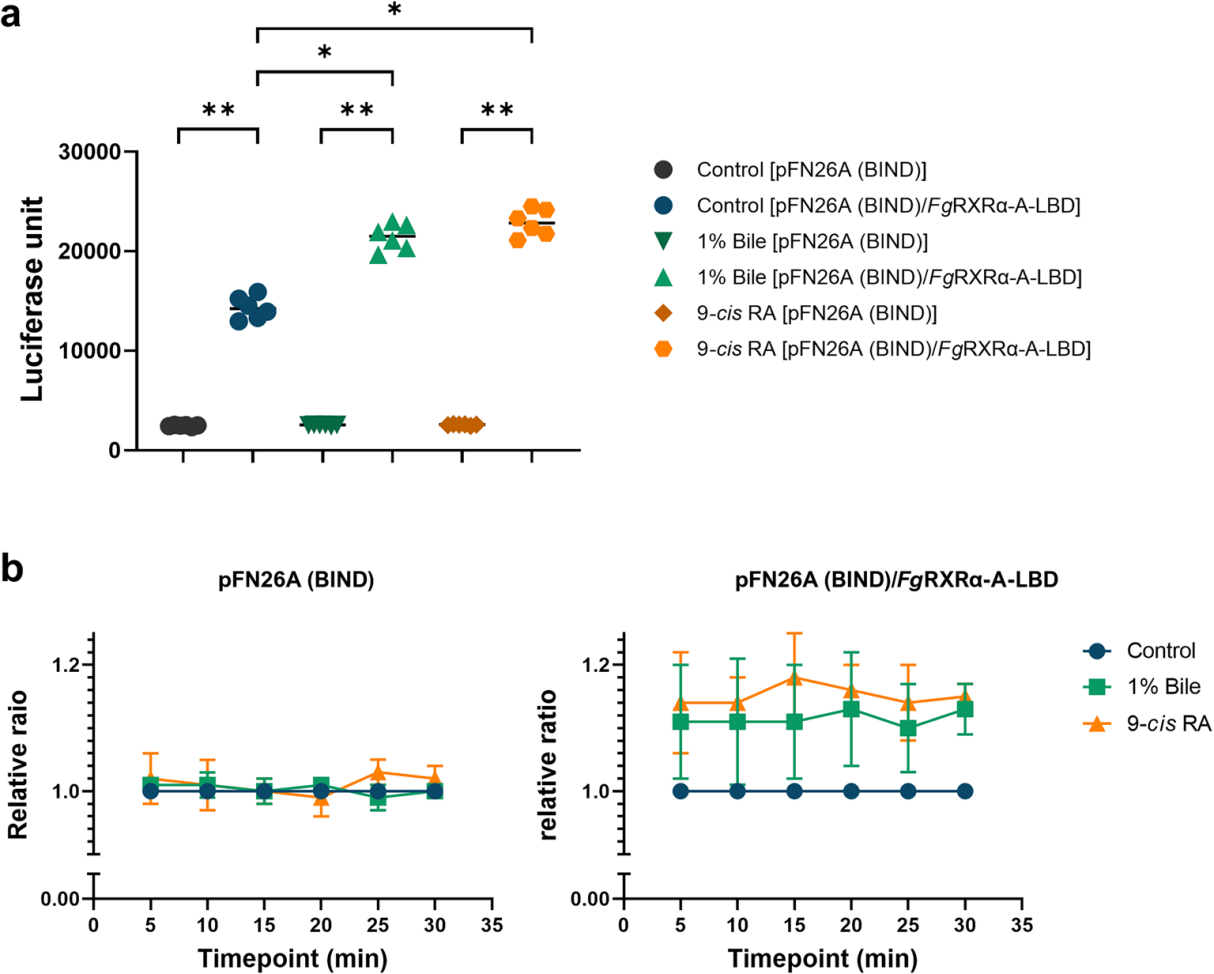
The binding assay of *Fg*RXRα-A-LBD with ligands. (**a**) The luciferase activities of pFN26A (BIND) and pFN26A (BIND)/ *Fg*RXRα-A-LBD when treated with 1% bile solution and 9-*cis* retinoic acid (RA) compared with untreated controls. (**b**) The relative ratios (mean ± SD) of pFN26A (BIND) and pFN26A (BIND)/ *Fg*RXRα-A-LBD when treated with 1% bile solution and 9-*cis* retinoic acid (RA) compared with untreated controls in a time-dependent manner. *p-value < 0.05 and **p-value < 0.001.

The functional assay of *Fg*RXRα-A was done by using the luciferase reporting system. The HEK293/pGL4.35[luc2P/9XGAL4UAS/Hygro] stable cells were transfected with pFN26A (BIND) hRluc-neo Flexi® vector with *Fg*RXRα-A-LBD or without insert. Both groups of transfected cells were treated with different ligands. The control groups of both transfected cells were cultured in the plain medium without ligand. The result demonstrated the baseline levels of luciferase signals from empty vector and pFN26A (BIND) hRluc-neo Flexi®/*Fg*RXRα-A-LBD transfected cells. The signal in the pFN26A (BIND) hRluc-neo Flexi®/*Fg*RXRα-A-LBD transfected cells was significantly higher than the pFN26A (BIND) empty vector (*p* < 0.001) (Fig. 5a). However, the luciferase signals in the pFN26A (BIND) hRluc-neo Flexi®/*Fg*RXRα-A-LBD transfected cells that treated with 1% bovine bile solution and 9-*cis* RA were significantly increased (*p* < 0.05) when compared with both empty vectors and untreated group (Fig. 5a). For the timepoint analysis, the relative ratios were calculated at 5, 10, 15, 20, 25, and 30 min after the reaction was stopped. The relative ratios were not different at all time points for the empty vector, pFN26A (BIND). In contrast, the 9-*cis* RA enhanced the luciferase activities in the pFN26A (BIND) hRluc-neo Flexi®/*Fg*RXRα-A-LBD transfected cells with the highest relative ratios at 15 min of 1.18 ± 0.07 (the relative ratios were ranging from 1.14 to 1.18). Moreover, the luciferase activities in the groups treated with 1% bile were also increased compared to the untreated control (the relative ratios ranged from 1.10 to 1.13) (Fig. 5b). The evaluation of the dose-dependent binding of 9-*cis* RA and *Fg*RXRα-A-LBD was done as shown in Fig. S3, and the binding assay result of the irrelevant ligand is shown in Fig. S3.

## Discussion

The complexity of the ecosystem, in which organisms live together, is very mysterious, particularly the parasitic relationship in which one organism gets benefit(s) from another organism^40^. *F. gigantica*, the platyhelminthic organism, is a true parasite that needs the hosts to complete their life cycle, including Lymnaeid snails and mammalian definitive hosts^5^. The adult stage of *F. gigantica* inhabits inside the liver of the definitive host and causes undesirable conditions^41^. From the past, we do not know why they need to travel from the intestine where they excysted to get maturation inside the liver and the large bile ducts^42,43^. Some reports suggest that the parasite needs some factors for their growth, such as nutrients and growth factors^41,43^. However, further study is required to support this hypothesis. Not only food cravings but sexual reproduction also occurs in the liver^44^. From this question, we tried to find the relationship between the molecules from the host that could interact with the parasite’s molecules and regulate their biology and physiology. Bile is a major accumulated component inside the liver and liver bile ducts^45^. Deeply, bile salts (the steroid-like molecule) are significant constituents of bile and, as such, are key molecules of interest because they will interact with the intracellular specific receptors, especially nuclear receptors (NRs), resulting in trigger downstream signaling cascades^46^.

Nuclear receptors (NRs) are the nucleoprotein family commonly known to have functions as transcription factors inside the nucleus of metazoans^45^. Many studies demonstrated that NRs involve several biological pathways, particularly regulating growth, development, differentiation, and reproduction when stimulated by the lipophilic hormones or molecules^47^. Hence, we are interested in the nuclear receptors (NRs) in *F. gigantica* according to its habitat and the potential to be an effective drug target. Our previous study reported the first characterized NR in *F. gigantica* (*Fg*NR1) that could be stimulated by bile solution^17^. The study on the deeper details of the *Fg*NR1 is still running, but simultaneously, we characterized the other NRs that would have a broader range of functions. Retinoid X receptor (RXR) was the selected target due to the interesting functions that, basically, it needs to form the heterodimer with other NRs such as thyroid receptor (TR), retinoic acid receptor (RAR), vitamin D receptor (VDR), farnesoid X receptor (FXR), liver X receptor (LXR), bile acid receptor (BAR), and peroxisome proliferator–activated receptors (PPARs). Moreover, the RXR can be from their own dimer for getting functions^22^.

The present study reported the first subfamily 2 nuclear receptor in *F. gigantica*, *Fg*RXRα-A. From bioinformatic analyses, it is suggested that the *Fg*RXRα-A DBD will bind to a similar DNA target as other RXRs using P-box, as it is highly conserved among the RXRs, which could be referred to as having similar downstream functions, providing strong evidence that *Fg*RXRα-A belongs to the RXR subfamily^20,48^. Whereas the D-box was 100% conserved among the other RXRs from flatworm parasites, it is less conserved among the RXRs of roundworms or mammals^48,49^. This result suggests that the interacting molecules (specifically other NRs) with *Fg*RXRα-A will be similar to those of other flatworms but different from those of other taxonomic organisms. This result is interesting because the unique properties could benefit drug development, which could target only parasites and not mammalian hosts. Moreover, it could be effective against a broad range of flatworms. However, the flatworm RXRs are not fully characterized, and most are reported only in databases from computational predictions^46,50–52^. Unlike DBD, LBD is more variable based on the specific ligands they interact with. However, *Fg*RXRα-A-LBD contains the signature sequence of NR-LBD (Tτ sequence)^49,53^. This finding suggested that the interacting ligands will be the same molecules or ligand family. Interestingly, *Fg*RXRα-A-LBD was exiguous conserved with RXRs from other isoforms and unrelated organisms. Phylogenetic analysis demonstrated similar results with multiple alignments. Our findings proved that the *Fg*RXRα-A is more closely related to *Fh*RXR and *Fb*RXRα than to other RXRs from flatworms, roundworms, mammals, and other isoforms. However, a limitation of this study is that the evolution of *Fg*RXRα-A cannot be definitely concluded because other members of the RXR family in* F. gigantica* are still missing. Moreover, the comparable analysis of the transcripts from closely related organisms, such as *Fh*RXR, could not be completed because of the unavailable gene expression profiles in the transcriptomic datasets. The unique characteristics of *Fg*RXRα-A are very attractive for drug discovery because it increases the chance of ligand selection, and the conservation among the flatworms could hopefully be a good target for several parasites at the same time.

Additionally, the 2D and 3D structures of *Fg*RXRα-A demonstrated the relative structure to the template nuclear receptor heterodimer. The 3D model revealed the two zinc finger domains that promptly bind to the target DNA. Meanwhile, the LBD demonstrated the specific binding pocket and surface ligands or potential therapeutic compounds may target. This information is valuable for designing targeted therapies and drug development efforts focused on modulating the activity of *Fg*RXRα-A. Additionally, the further computational analysis of the *Fg*RXRα-A 3D structure can facilitate the virtual screening of compound libraries to identify potential ligands as well as inhibitors that could modulate *Fg*RXRα-A activity, paving the way for the development of novel therapeutic agents^21,45^.

The Western analysis revealed that the native *Fg*RXRα-A could be detected in the insoluble fraction of the crude worm antigens (CWA-I) at the expected size (60 kDa). This result confirmed that the *Fg*RXRα-A could be found in adult *F. gigantica*. Interestingly, the native *Fg*RXRα-A can be localized concentratedly in the testes of the adult worm. It is correlated with our previous study of *Fg*NR1, which was detected in the testes and tips of caeca^17^. As previously known, the NRs can interact with several sex hormones, such as testosterone and estrogen, and facilitate sexual development^54–56^. These findings provided strong evidence that activated *Fg*RXRα-A could be related to testes functions. Hence, we hypothesized that *Fg*RXRα-A would be critical molecules for the sexual reproduction of the parasite. However, this study was performed only in adult parasites, so the function of *Fg*RXRα-A in reproductive maturation could not be confirmed. Nevertheless, based on previous studies of RXRs in other organisms, we proposed that FgRXRα-A plays a role in the maturation of reproductive organs. Hence, if drugs are discovered to interrupt the maturation of the reproductive organs, the parasite will not develop into the adult stage, resulting in a reduction of severe complications^14,54^.

The functional study of *Fg*RXRα-A was done using the modified binding assay of the estrogen receptor^57^. Our findings suggested that 9-*cis* RA could bind specifically to *Fg*RXRα-A-LBD. However, as shown in Supplementary Fig. S3, the dose-specificity of the receptor was not revealed by 9-*cis* RA, although the selectivity in the binding of chemically different ligands was shown. It is the confirmation that sequence conservation and *Fg*RXRα-A may have the same function as other RXRs by triggering the downstream signaling cascades throughout the development in the vertebrate host as expected in *Schistosoma mansoni*^51^. Interestingly, our result demonstrated that the bile solution can activate the *Fg*RXRα-A function by increasing luciferase activity. This result suggested that bile contains some molecules that could be the ligand for *Fg*RXRα-A that will be further identified. However, the background in the cells transfected with pFN26A (BIND) containing *Fg*RXRα-A-LBD was higher than in the empty vector control, pFN26A (BIND). This finding suggests that some molecules accumulating in the culture media can bind to *Fg*RXRα-A-LBD and trigger downstream luciferase genes, resulting in much higher luciferase units than the empty plasmid. However, the signal was still lower than that of the pFN26A (BIND)/*Fg*RXRα-A-LBD transfected cells treated with a specific ligand (9-*cis* RA) as well as a bile solution. This study provides the assumption that *F. gigantica* needs some molecules, such as steroid-like molecules, from the host for their living, maturation, and sexual development, and the hormone-specific receptors in the parasite could be the potential target for novel drug development. Nevertheless, ligand trafficking and intracellular signal transduction still need to be explored.

## Conclusion

In conclusion, this study first identified and characterized the retinoid X receptor (RXR) in *F. gigantica* (*Fg*RXRα-A). *Fg*RXRα-A demonstrated the conserved sequence to the other RXRs at both DBD and LBD domains, especially to the RXRs from the flatworms. Moreover, *Fg*RXRα-A was successfully cloned and produced as a recombinant protein that let us study in more detail by producing polyclonal antibodies. Interestingly, *Fg*RXRα-A was detected in the crude extract of adult parasite and highly expressed in the testes of adult worms. Additionally, *Fg*RXRα-A could be activated by 9-*cis* RA, which is the specific ligand for any RXRs. Our study not only provides important biological data on the parasite that interacts with the host’s molecules through nuclear receptors, but our results suggest that *Fg*RXRα-A could be a potential target for fascioliasis drugs. However, the specific ligands in the bile components and the expression of *Fg*RXRα-A in other developmental stages are still required to fully understand the *Fg*RXRα-A functions that will be useful for drug discovery in the future.

### Supplementary Information

Supplementary Information 1.

Supplementary Information 2.

Supplementary Information 3.

Supplementary Information 4.

Supplementary Information 5.

Supplementary Information 6.

## Data Availability

The datasets generated during and/or analyzed during the current study are available from the corresponding author upon reasonable request.
