# Supplementary figures and images for: Molecular and biochemical characterizations of a Fasciola gigantica retinoid X receptor-α isoform A (FgRXRα-A)
Source: Sci Rep. 2024 May 29;14:12347. doi: 10.1038/s41598-024-63194-6 (PMC11137005; doi:10.1038/s41598-024-63194-6)

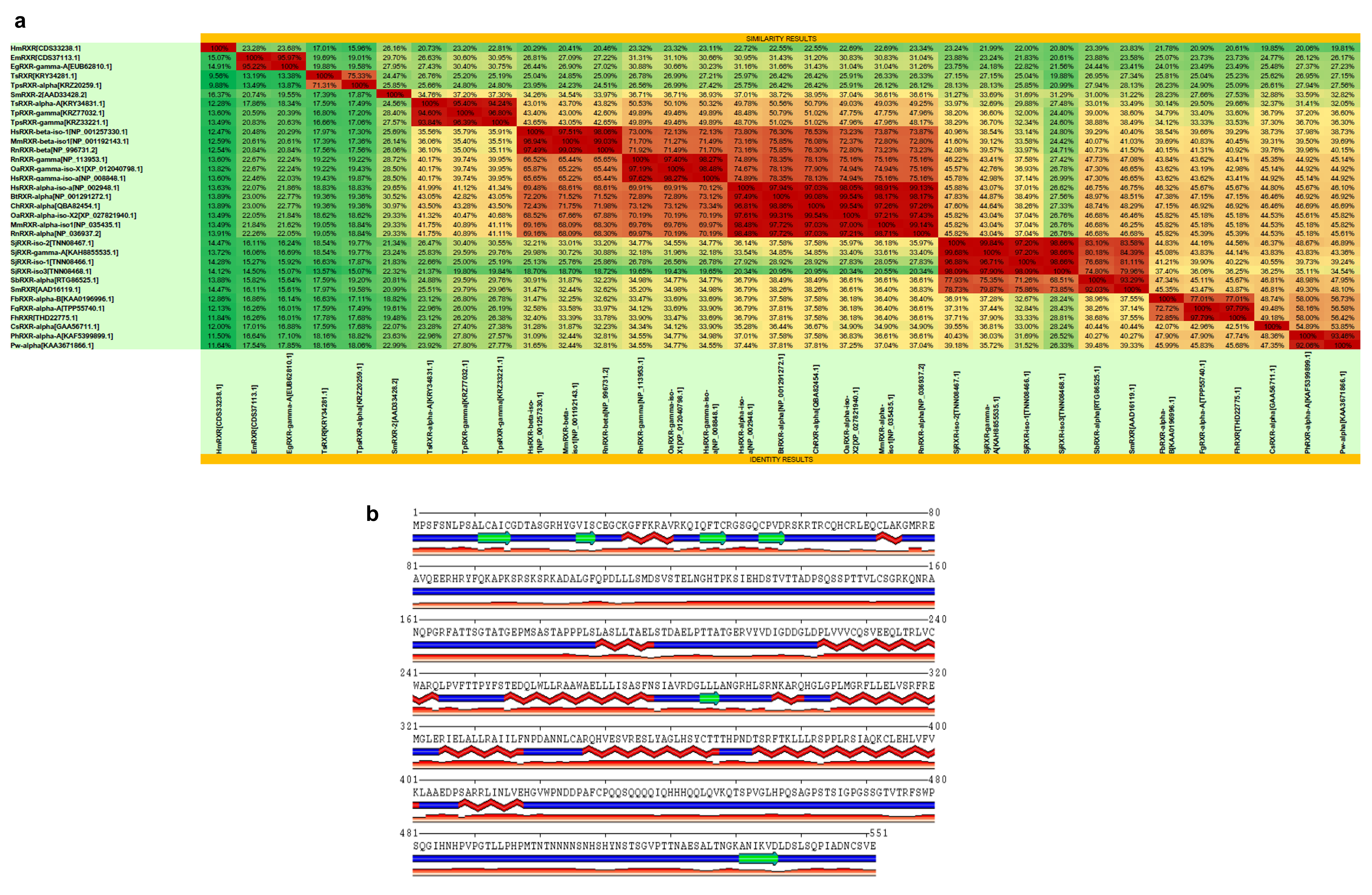

Supplement: Supplementary file 1 — Supplementary Information 1. [file 41598_2024_63194_MOESM1_ESM.png]

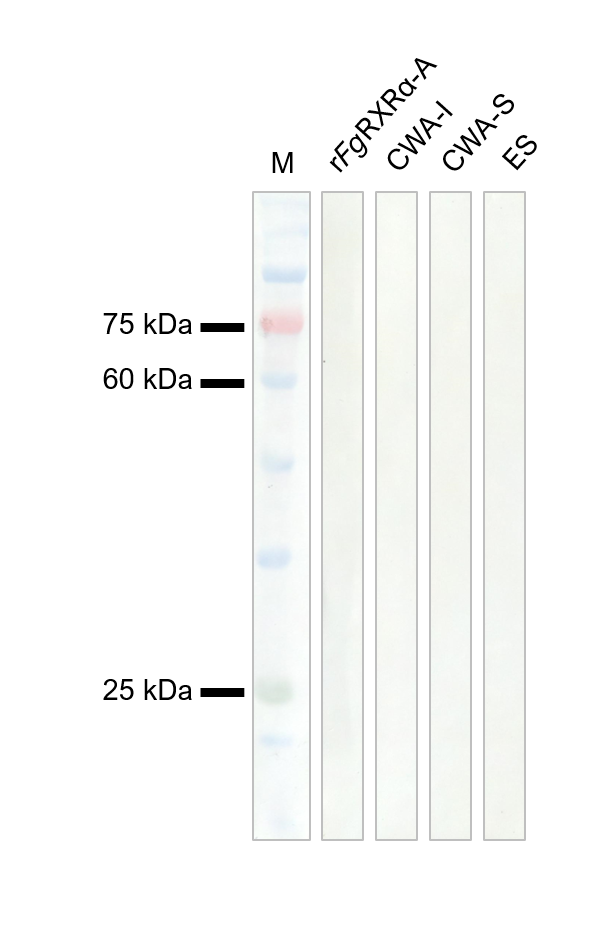

Supplement: Supplementary file 2 — Supplementary Information 2. [file 41598_2024_63194_MOESM2_ESM.png]

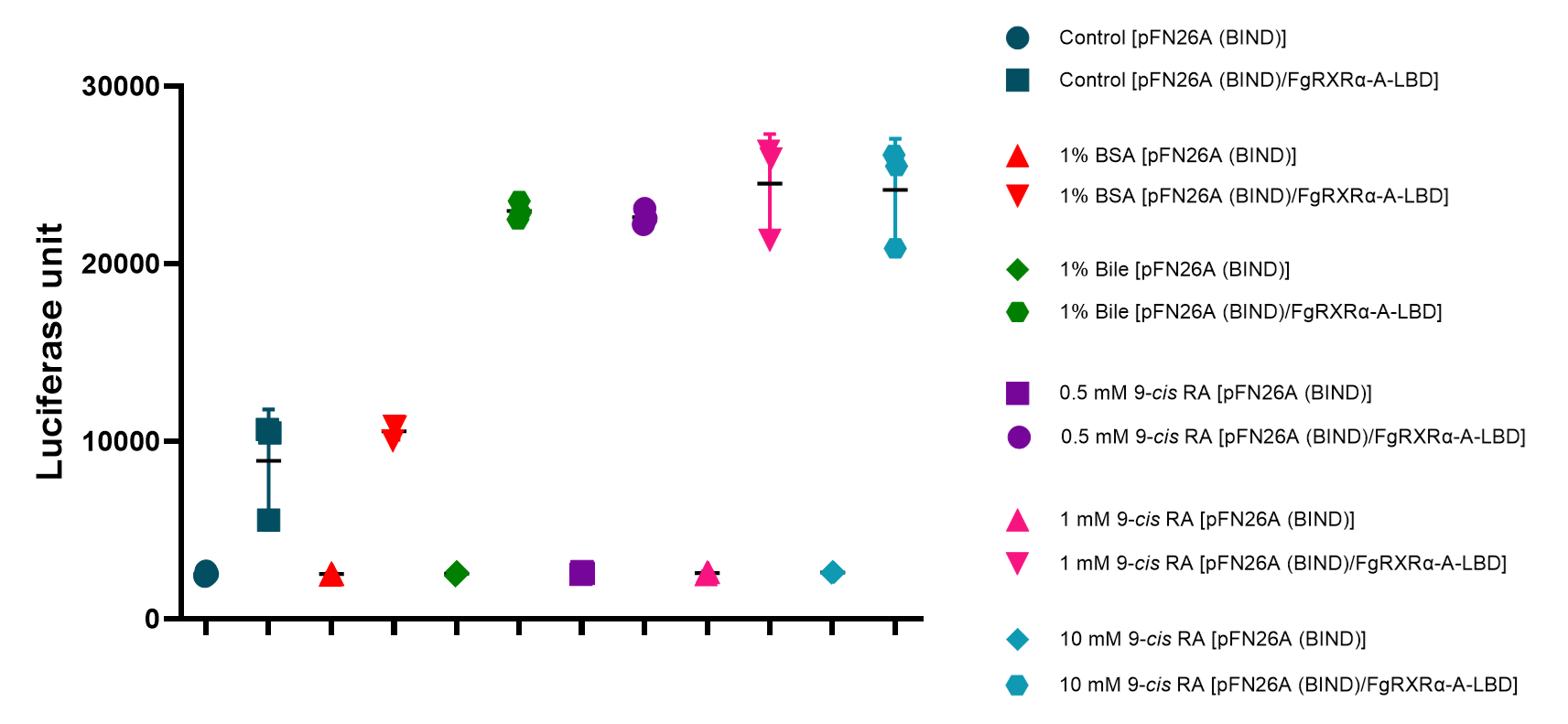

Supplement: Supplementary file 3 — Supplementary Information 3. [file 41598_2024_63194_MOESM3_ESM.png]
