# Supplementary material for: Molecular and biochemical characterizations of a Fasciola gigantica retinoid X receptor-α isoform A (FgRXRα-A)
Source: Sci Rep. 2024 May 29;14:12347. doi: 10.1038/s41598-024-63194-6 (PMC11137005; doi:10.1038/s41598-024-63194-6)

<u>**Gels and blots used in this manuscript**</u>

Figure 2a

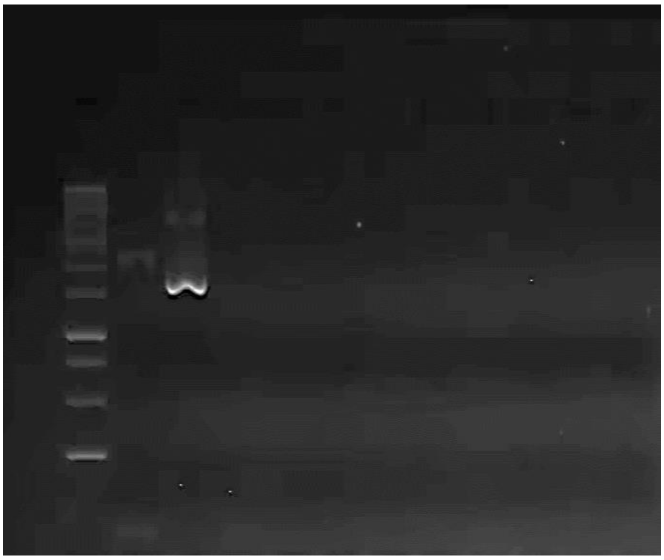

Figure 2b

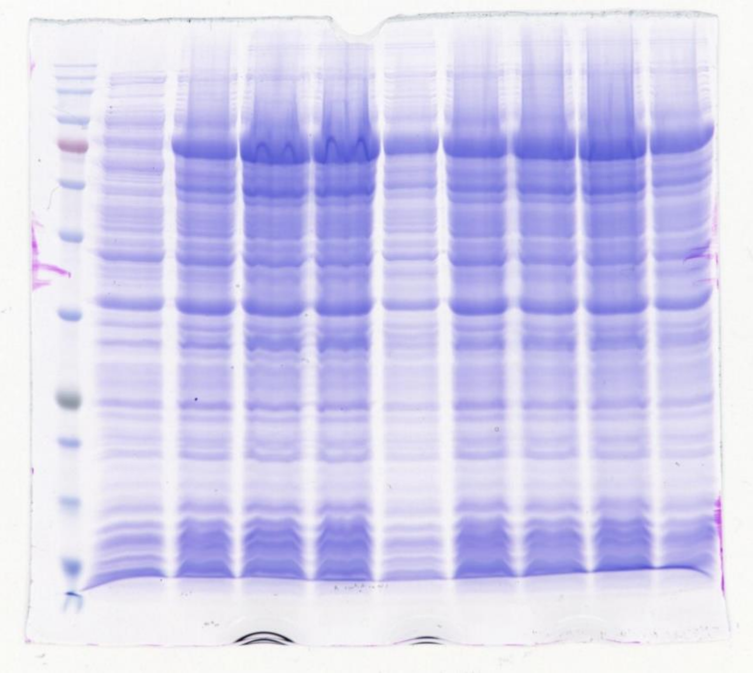

Figure 2c

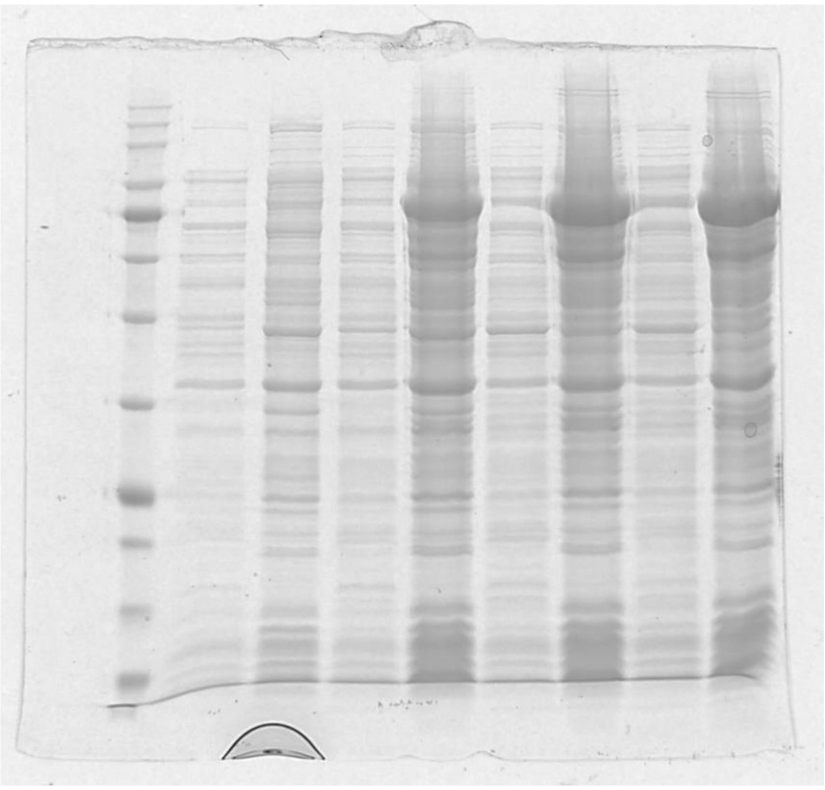

Figure 2d

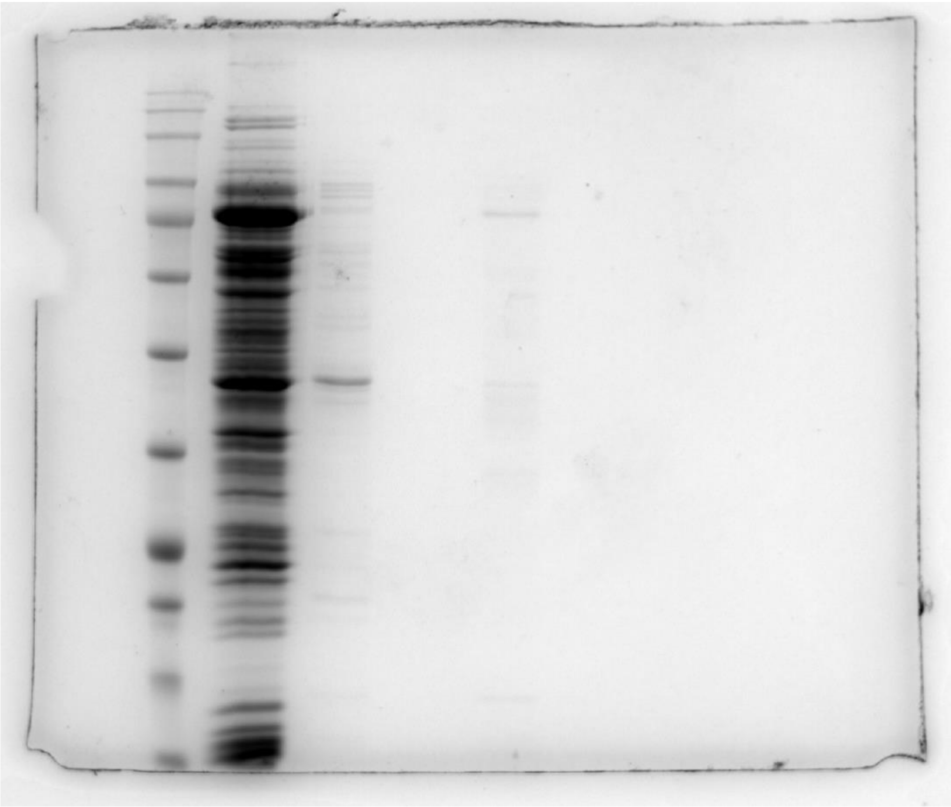

Figure 2e

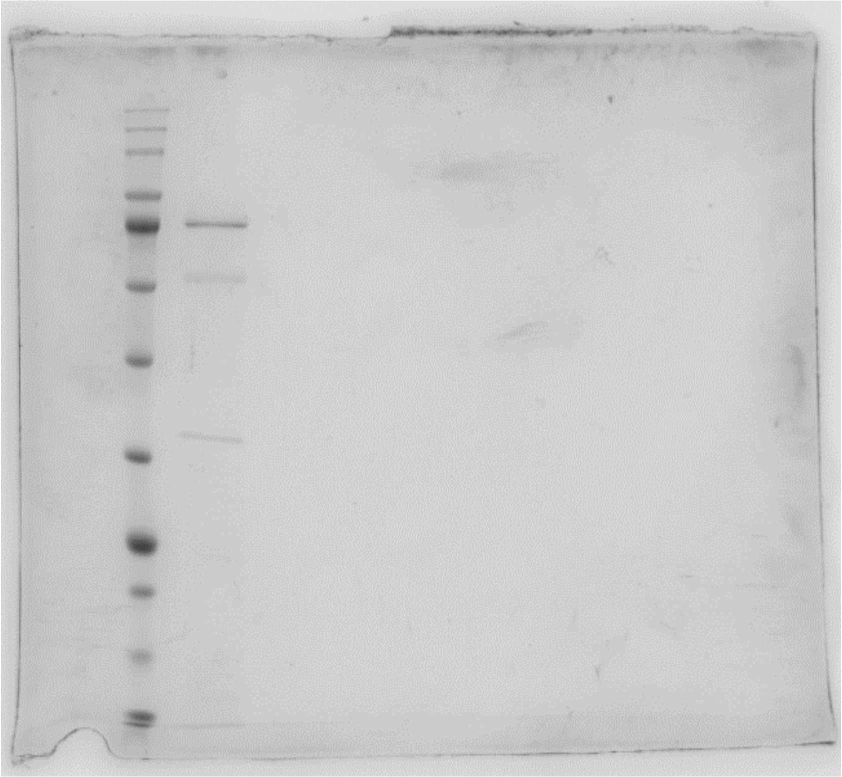

Figure 3a

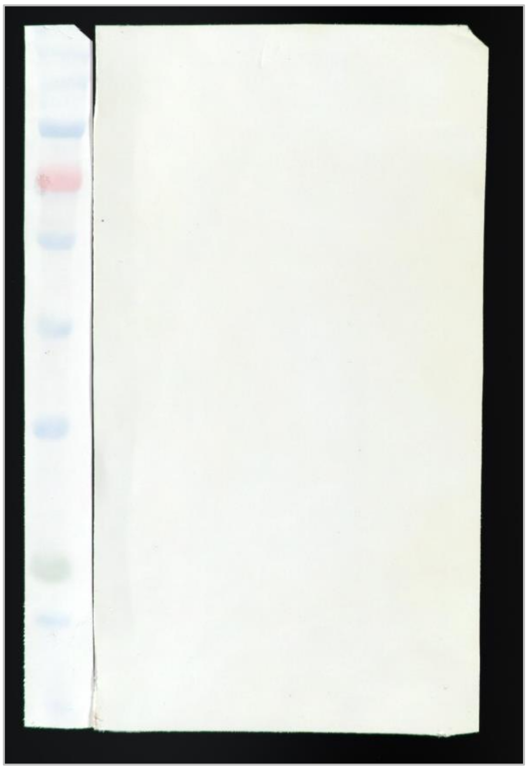

Figure 3c (left)

Figure 3b (left)

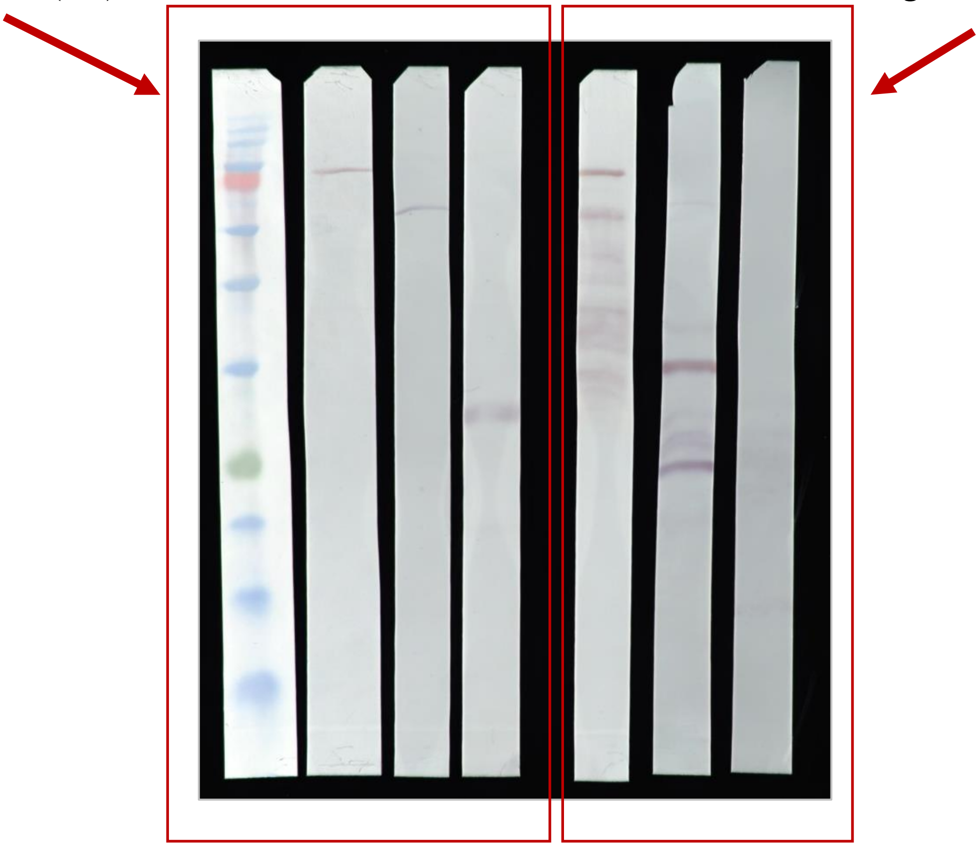

Figure 3b (right)

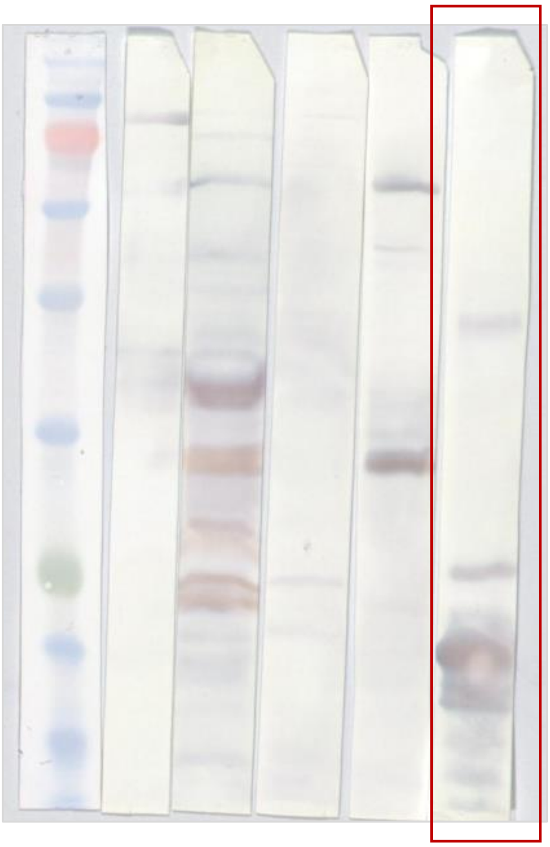

Figure 3c (right)

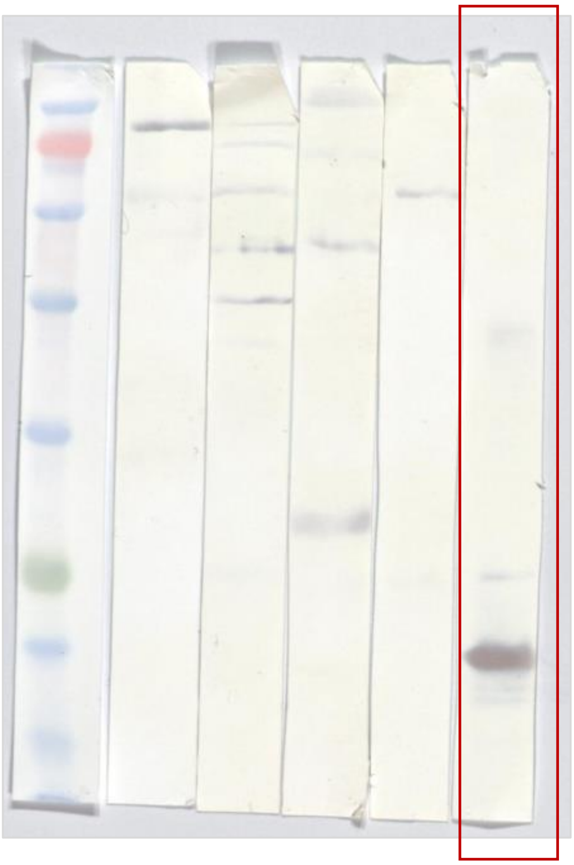

Supplement: Supplementary file 4 — Supplementary Information 4. [file 41598_2024_63194_MOESM4_ESM.pdf]
