# Supplementary material for: Molecular and biochemical characterizations of a Fasciola gigantica retinoid X receptor-α isoform A (FgRXRα-A)
Source: Sci Rep. 2024 May 29;14:12347. doi: 10.1038/s41598-024-63194-6 (PMC11137005; doi:10.1038/s41598-024-63194-6)

**Figure legends (Supplementary figures)**

**Figure S1:** (**a**) Heatmap analysis of *Fg*RXRα-A with other homologs shows the highest conservation of *Fg*RXRα-A with *Fb*RXRα-B and *Fh*RXR (black box). The red color represents high conservation, followed by orange, dark yellow, yellow, light green, and dark green in descending order. (**b**) The 2D structure of *Fg*RXRα-A, green arrows represent the β-sheet, and red zigzag patterns represent the α-helices.

**Figure S2:** Western analysis of *F. gigantica* crude worm antigen soluble fraction (CWA-I), crude worm antigen insoluble fraction (CWA-S), excretory/secretory products (ES), and r*Fg*RXRα-A with preimmune sera.

**Figure S3:** The optimization of binding assay with ligands including control (without ligand), irrelevant ligand (1% bovine serum albumin; BSA), 1% bile solution, 0.5 mM, 1 mM, and 10 mM 9-*cis* RA in the pFN26A (BIND) and pFN26A (BIND)/ *Fg*RXRα-A-LBD transfected HEK293 cells.


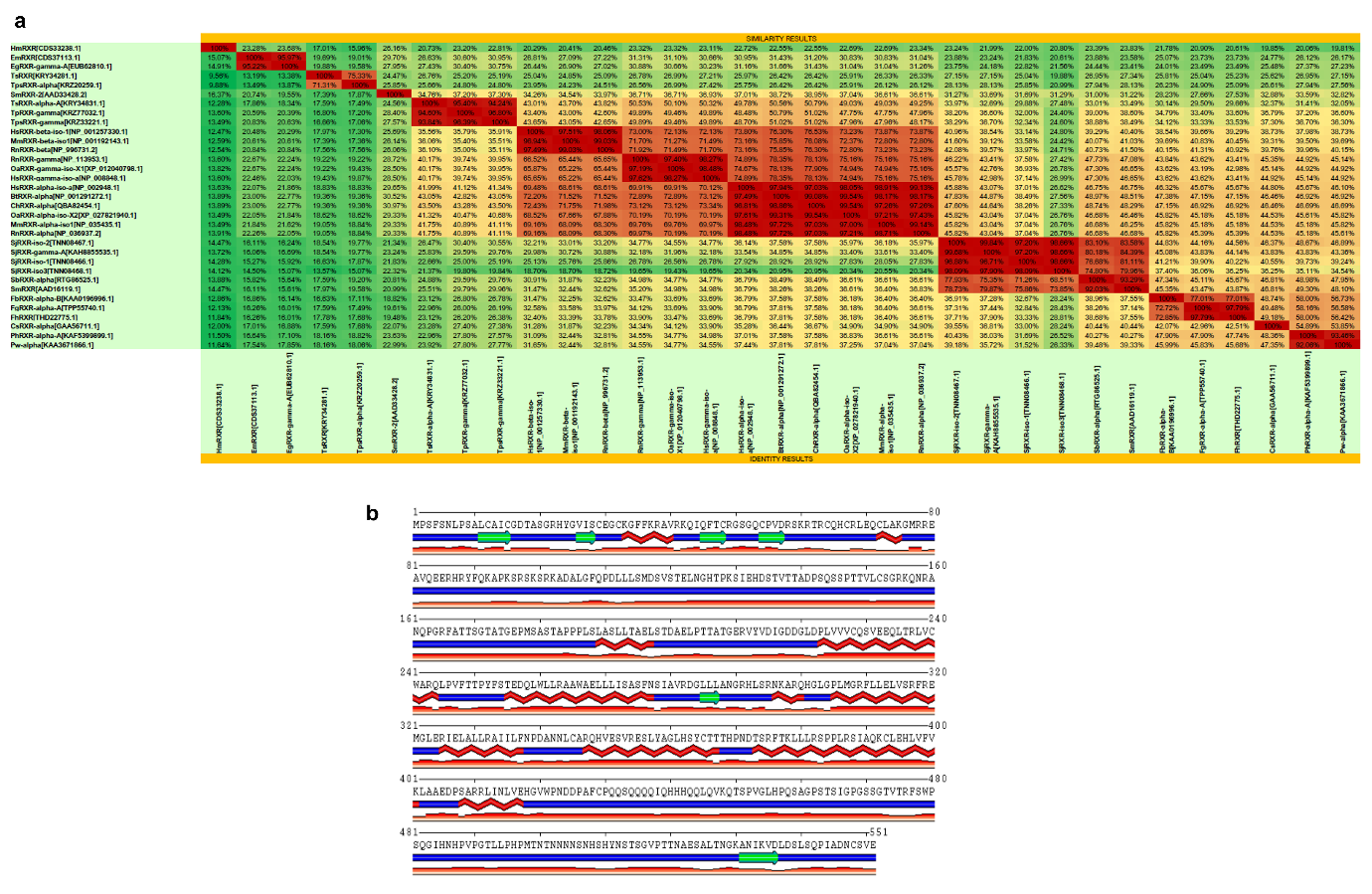


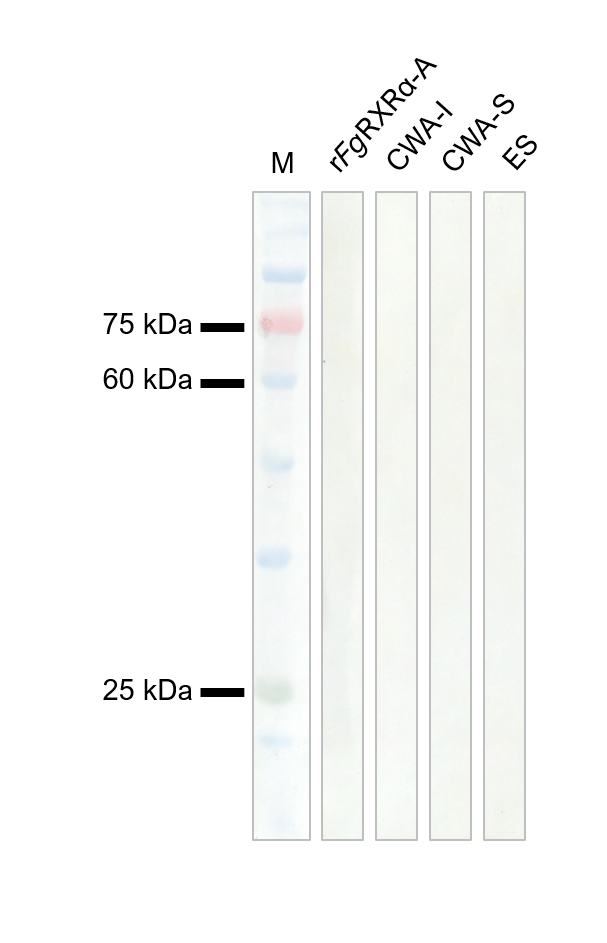


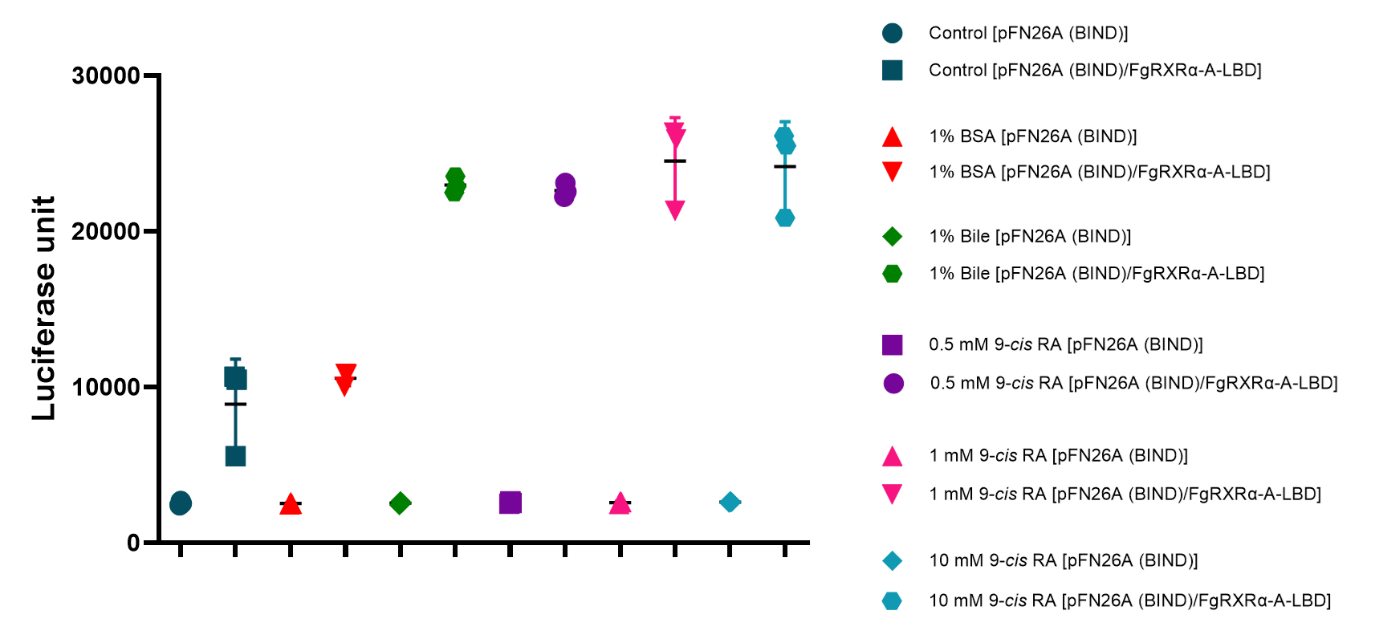

Supplement: Supplementary file 5 — Supplementary Information 5. [file 41598_2024_63194_MOESM5_ESM.docx]
