# Supplementary material for: Molecular and biochemical characterizations of a Fasciola gigantica retinoid X receptor-α isoform A (FgRXRα-A)
Source: Sci Rep. 2024 May 29;14:12347. doi: 10.1038/s41598-024-63194-6 (PMC11137005; doi:10.1038/s41598-024-63194-6)
Supplement: Supplementary file 6 — Supplementary Information 6. [file 41598_2024_63194_MOESM6_ESM.docx]

**Table S1:** Abbreviations and GenBank/accession numbers of RXR homologs used in this study.

| **Abbreviation** | **Species** | **Protein name** | **GenBank/ Accession no.** |
| --- | --- | --- | --- |
| BtRXRα | *Bos taurus* | Retinoic acid receptor RXR-alpha | NP_00191272.1 |
| ChRXRα | *Capra hircus* | Retinoic acid receptor RXR-alpha | QBA82454.1 |
| CsRXR | *Clonorchis sinensis* | Retinoic acid receptor RXR | KAG5442315.1 |
| CsRXRα | *Clonorchis sinensis* | Retinoic acid receptor RXR-alpha | GAA56711.1 |
| CsRXRɤ | *Clonorchis sinensis* | Retinoic acid receptor RXR-gamma | GAA29255.2 |
| EmRXR | *Echinococcus multilocularis* | Retinoic acid receptor RXR | CDS37113.1 |
| FbRXRα-B | *Fasciolopsis buski* | Retinoid x receptor alpha B | KAA0196996.1 |
| FgRXRα-A | *Fasciola gigantica* | Retinoic acid receptor RXR-alpha-A | TPP55740.1 |
| FhRXR | *Fasciola hepatica* | Retinoic acid receptor RXR | THD22775.1 |
| HmRXR | *Hymenolepis microstoma* | Retinoic acid receptor RXR | CDS33238.1 |
| HsRXRα-A | *Homo sapiens* | Retinoic acid receptor RXR-alpha isoform a | NP_002948.1 |
| HsRXRβ-1 | *Homo sapiens* | Retinoic acid receptor RXR-beta isoform 1 | NP_001257330.1 |
| HsRXRɤ-A | *Homo sapiens* | Retinoic acid receptor RXR-gamma isoform a | NP_008848.1 |
| MmRXRα-1 | *Mus musculus* | Retinoic acid receptor RXR-alpha isoform 1 | NP_035435.1 |
| MmRXRβ-1 | *Mus musculus* | Retinoic acid receptor RXR-beta isoform 1 | NP_001192143.1 |
| OaRXRα-X1 | *Ovis aries* | Retinoic acid receptor RXR-alpha isoform X1 | XP_027821940.1 |
| OaRXRɤ-X1 | *Ovis aries* | Retinoic acid receptor RXR-gamma isoform X1 | XP_012040798.1 |
| PhRXRα-A | *Paragonimus heterotremus* | Retinoic acid receptor RXR-alpha-A | KAF5399899.1 |
| PwRXRα | *Paragonimus westermani* | Retinoic acid receptor RXR-alpha | KAA3671866.1 |
| RnRXRα | *Rattus norvegicus* | Retinoic acid receptor RXR-alpha | NP_036937.2 |
| RnRXRβ | *Rattus norvegicus* | Retinoic acid receptor RXR-beta | NP_113953.1 |
| RnRXRɤ | *Rattus norvegicus* | Retinoic acid receptor RXR-gamma | NP_996731.2 |
| SbRXRα | *Schistosoma bovis* | Retinoic acid receptor RXR-alpha | RTG86525.1 |
| SjRXR | *Schistosoma japonicum* | Retinoic acid receptor RXR | KAH8855537.1 |
| SjRXR1 | *Schistosoma japonicum* | Retinoic acid receptor RXR isoform 1 | TNN08466.1 |
| RjRXR2 | *Schistosoma japonicum* | Retinoic acid receptor RXR isoform 2 | TNN08467.1 |
| SjRXR3 | *Schistosoma japonicum* | Retinoic acid receptor RXR isoform 3 | TNN08468.1 |
| SjRXRɤ-A | *Schistosoma japonicum* | Retinoic acid receptor RXR-gamma-A | KAH8855535.1 |
| SmRXR | *Schistosoma mansoni* | Retinoic acid receptor RXR | XP_018645908.1 |
| TbRXR | *Trichinella britovi* | Retinoic acid receptor RXR | KRY51200.1 |
| TmRXR | *Trichinella murrelli* | Retinoic acid receptor RXR | KRX40980.1 |
| TnRXR | *Trichinella nelson* | Retinoic acid receptor RXR | KRX26289.1 |
| TpRXR | *Trichinella patagoniensis* | Retinoic acid receptor RXR | KRY11544.1 |
| TsRXRα | *Trichinella spiralis* | Retinoic acid receptor RXR-alpha | XP_003379573.1 |
| TsRXRα-A | *Trichinella spiralis* | Retinoic acid receptor RXR-alpha-A | KRY34831.1 |

**Table S2:** Molecular characteristics of FgRXRα

| **Characteristics** | **Result** | **Predictive program** |
| --- | --- | --- |
| **Residues** | 511 amino acids | EMBOSS Pepstats |
| **Molecular weight** | 60.355 kDa |  |
| **Isoelectric Point** | 8.3669 |  |
| **Signal peptide** | No | SignalP 5.0 |
| **Transmembrane** | No | TMHMM 2.0 |
| **N-glycosylation sites** | N_371_, N_504_, N_507_, N_512_, N_547_ | NetNGlyc 1.0 |
| **O-glycosylation sites** | S_3_, S_5_, S_69_, S_98_, S_100_, S_115_, S_118_, S_120_, T_121_, T_127_, S_130_, S_135_, S_136_, T_138_, T_139_, S_143_, S_145_, S_146_, T_148_, T_149_, S_153_, T_168_, T_169_, S_170_, T_172_, T_174_, S_179_, S_181_, T_182_, S_188_, S_191_, S_198_, T_199_, T_205_, T_206_, T_208_, T_250_, S_433_, T_450_, S_451_, S_459_, S_463_, T_464_, S_465_, S_470_, S_471_, T_473_, T_475_, S_478_, S_481_, T_492_, T_499_, T_501_, S_506_, S_506_, S_509_, S_513_, T_514_, S_515_, T_519_, T_520_, S_524_ | NetOGlyc 4.0 |
| **Predicted disulfide bonds** | Total number of cysteines: 17  Predicted number of bonds: 6  Bond index  12-29, 48-64, 15-32, 228-240,  54-67, and 345-365 | SCRATCH Protein Predictor |
